# Effect of mobile interventions with nicotine replacement therapy sampling on long-term smoking cessation in community smokers: A pragmatic randomized clinical trial

**DOI:** 10.18332/tid/160168

**Published:** 2023-03-24

**Authors:** Ningyuan Guo, Tzu Tsun Luk, Yongda Socrates Wu, Ziqiu Guo, Jessica Chi Lok Chu, Yee Tak Derek Cheung, Ching Han Helen Chan, Tyrone Tai On Kwok, Victor Yiu Lun Wong, Carlos King Ho Wong, Jung Jae Lee, Yu Kwong Kwok, Kasisomayajula Viswanath, Tai Hing Lam, Man Ping Wang

**Affiliations:** 1 School of Nursing, Shanghai Jiao Tong University, Shanghai, China; 2 School of Nursing, The University of Hong Kong, Hong Kong, China; 3 Tung Wah Group of Hospitals Integrated Centre on Smoking Cessation, Hong Kong, China; 4 Technology-Enriched Learning Initiative, The University of Hong Kong, Hong Kong, China; 5 Department of Family Medicine and Primary Care, School of Clinical Medicine, The University of Hong Kong, Hong Kong, China; 6 Department of Pharmacology and Pharmacy, The University of Hong Kong, Hong Kong, China; 7 Laboratory of Data Discovery for Health, Hong Kong Science and Technology Park, Hong Kong, China; 8 School of Science and Technology, Hong Kong Metropolitan University, Hong Kong, China; 9 Center for Community-Based Research, Dana-Farber Cancer Institute, Boston, United States; 10 Department of Social and Behavioral Sciences, T.H. Chan School of Public Health, Harvard University, Boston, United States; 11 School of Public Health, The University of Hong Kong, Hong Kong, China

**Keywords:** smoking cessation, randomized controlled trial, mHealth, community smoker, messaging

## Abstract

**INTRODUCTION:**

Mobile interventions enable personalized behavioral support that could improve smoking cessation (SC) in smokers ready to quit. Scalable interventions, including unmotivated smokers, are needed. We evaluated the effect of personalized behavioral support through mobile interventions plus nicotine replacement therapy sampling (NRT-S) on SC in Hong Kong community smokers.

**METHODS:**

A total of 664 adult daily cigarette smokers (74.4% male, 51.7% not ready to quit in 30 days) were proactively recruited from smoking hotspots and individually randomized (1:1) to the intervention and control groups (each, n=332). Both groups received brief advice and active referral to SC services. The intervention group received 1-week NRT-S at baseline and 12-week personalized behavioral support through SC advisor-delivered Instant Messaging (IM) and a fully automated chatbot. The control group received regular text messages regarding general health at a similar frequency. Primary outcomes were carbon monoxide-validated smoking abstinence at 6 and 12 months post-treatment initiation. Secondary outcomes included self-reported 7-day point-prevalence and 24-week continuous abstinence, quit attempts, smoking reduction, and SC service use at 6 and 12 months.

**RESULTS:**

By intention-to-treat, the intervention group did not significantly increase validated abstinence at 6 months (3.9% vs 3.0%, OR=1.31; 95% CI: 0.57–3.04) and 12 months (5.4% vs 4.5%, OR=1.21; 95% CI: 0.60–2.45), as were self-reported 7-day point-prevalence abstinence, smoking reduction, and SC service use at 6 and 12 months. More participants in the intervention than control group made a quit attempt by 6 months (47.0% vs 38.0%, OR=1.45; 95% CI: 1.06–1.97). Intervention engagement rates were low, but engagement in IM alone or combined with chatbot showed higher abstinence at 6 months (adjusted odds ratios, AORs=4.71 and 8.95, both p<0.05).

**CONCLUSIONS:**

Personalized behavioral support through mobile interventions plus NRT-S did not significantly improve abstinence in community smokers compared to text only messaging. The suboptimal intervention engagement needs to be addressed in future studies.

**TRIAL REGISTRATION:**

ClinicalTrials.gov NCT04001972.

## INTRODUCTION

Mobile interventions enable highly accessible, lowcost, and personalized behavioral support for smoking cessation (SC). A Cochrane review of 13 randomized controlled trials (RCTs) showed that text messaging interventions through Short Message Service (SMS) were more effective than minimal SC support in increasing quitting (risk ratio=1.54; 95% CI: 1.19–2.00) at 6 months or longer (11 at 6 months; 2 at 12 months)^1^. Longer term effect of mobile SC interventions is uncertain as few RCTs (15%) had a follow-up beyond 6 months^1,2^. Most trials targeted smokers willing to make a quit attempt in the next 30 days^3-5^. The population-level effect of mobile interventions remains unknown as many who do not want to quit or plan to quit were not included. Over two thirds (68.8% in 2019) of current smokers had never made a quit attempt in Hong Kong, where the smoking prevalence has been one of the lowest worldwide (10.2% in 2019)^6^. Mobile interventions in the community smokers with longer follow-up length are needed.

Instant Messaging (IM, e.g. WhatsApp, WeChat) is a popular and inexpensive alternative to SMS. Our qualitative interviews on community smokers (76% had no quit plan in the next 6 months) showed that the provision of more personalized behavioral support from human SC advisors was the most valued utility of IM for SC^7^. Our pragmatic RCT further showed that IM intervention was effective for SC in community smokers^8^. However, the intervention engagement rate was low (17%), which might be due to the unavailability of human SC advisors outside office hours^8^. SC support could be sustained using chatbots (also known as conversational agents), online computer programs that can simulate human conversations. Evidence on chatbots for SC is emerging but remains scarce and limited. A formative study showed that a chatbot increased motivation to quit immediately after usage in a volunteer sample of young smokers^9^. An RCT focusing on smokers motivated to quit identified that adding a chatbot to an SC app more than doubled intervention engagement with the app (incidence rate ratios=2.01; 95% CI: 1.92–2.11), but the effect on SC was unclear because of a low retention rate at 1 month (10.7%)^10^. A pragmatic RCT in primary care settings showed that a chatbot was marginally more effective than usual care (biochemically validated abstinence at 6 months: OR=1.52; 95% CI: 1.00–2.31; p=0.05) despite of potential non-response bias due to a low retention rate (45.2%)^11^.

The interventions in the present RCT were developed based on established evidence of our prior studies and RCTs. We have developed and tested an approach^12^ of proactively reaching community smokers who were largely unmotivated to quit and reasonably representative of the general smoking population regarding their sociodemographic and smoking characteristics^8^. Our 2015 RCT in the proactively recruited community smokers showed that brief advice using the AWARD model (Ask, Warning, Advice, Referral, Do-it-again) was effective for SC^13^. Our 2017 RCT further developed the IM intervention combined with the AWARD model and supported the effectiveness for SC^8^. Nicotine replacement therapy sampling (NRT-S) has been used in unmotivated smokers and was found to be effective for increasing quit attempts and full-course NRT use^14^. A recent RCT in unmotivated smokers showed that mobile intervention plus NRT-S led to higher abstinence at 6 months than NRT-S alone^15^. Our pilot RCT showed that the IM intervention plus NRT-S was feasible with positive effects on quitting, smoking reduction, quit attempts, and NRT-S use in community smokers^16^. The present RCT developed a chatbot in addition to the established AWARD model, IM intervention, and NRT-S. We aimed to evaluate the long-term (6 and 12 months) effect of personalized behavioral support through IM and chatbot plus nicotine replacement therapy sampling (NRT-S) on SC in Hong Kong community smokers.

## METHODS

### Study design

This was a two-arm, parallel, assessor-blinded randomized controlled trial that was fairly pragmatic according to PRECIS-2 (Pragmatic Explanatory Continuum Indicator Summary) tool^17^. We followed the Consolidated Standards of Reporting Trials of Electronic (CONSORT)-EHEALTH extension (Supplementary file)^18^. The trial protocol is available in the Supplementary file. All participants provided written informed consent.

### Setting and participants

Participants were proactively recruited from smoking hotspots, outdoor places where smokers gather and smoke (e.g. exits of underground transit and railway stations, shopping malls, and large commercial buildings), throughout Hong Kong from 19 August 2019 to 8 May 2020. Note that the recruitment was temporally suspended from 22 January 2020 to 9 April 2020 due to the COVID-19 pandemic (n=623 recruited pre-COVID; n=41 recruited post-COVID). We recruited Hong Kong residents aged ≥18 years who were able to read and communicate in Chinese; currently smoked at least one cigarette daily, validated by an exhaled carbon monoxide level of ≥4 parts per million (ppm); and owned a smartphone and were willing to install an IM app (if not already installed). The inclusion criteria of smoking at least one cigarette daily was consistently used in our prior trials for recruiting community smokers who were largely representative of daily cigarette smokers in the general population in Hong Kong^8,13,16^. Smoking at least once cigarette daily has also been widely used for defining daily smokers in large-scale studies worldwide^19,20^. Exclusion criteria included smokers who had psychiatric/psychological diseases or were on regular psychotropic medications; were using SC medication, NRT, other SC services; or had contraindication for NRT use, including severe angina, arrhythmia, myocardial infarction, pregnancy (or intended to become pregnant within 6 months) or breastfeeding, which were screened using dichotomous questions (yes/no).

An experienced research assistant and trained SC advisors proactively approached smokers using a ‘foot-in-the-door’ approach. Smokers were asked about smoking behaviors, assessed for exhaled carbon monoxide level, and invited to participate in the study. Those showing interest were assessed for eligibility, and written informed consent was sought. Eligible participants completed a brief self-administered baseline questionnaire to provide data on sociodemographic and smoking characteristics and quality of life. To avoid intervention contamination, 1 smoker was randomly approached when there were more smokers at the same hotspot. Smoking-related outcomes were measured in follow-up questionnaires through telephone interviews conducted by the experienced research assistant or trained SC advisors at 3, 6, and 12 months after randomization (intervention initiation), and quality of life was additionally assessed at 12 months.

### Randomization and masking

The randomization sequence with a 1:1 allocation ratio and permuted block of 4, 8, or 12 was generated by a non-investigator. Sequentially numbered, opaque, sealed envelopes (SNOSE) were prepared by an investigator not involved in participant enrolment for allocation concealment. Once a smoker signed the consent form, one SNOSE was opened according to the serial number to determine the group allocation. Masking of participants, the research assistant, and SC advisors was not possible due to the nature of behavioral interventions. Statistical analysts were blinded from the group allocation.

### Interventions

Both groups received brief advice using the AWARD model (Ask, Warning, Advice, Referral, Do-it-again) at baseline^13^. Participants were asked about smoking behaviors (Ask) and invited for an exhaled carbon monoxide test. The results were used to warn about the harms of continued smoking together with a leaflet (Supplementary file Figure 1) containing shocking pictures of smoking-induced diseases (Warn). Participants were advised to quit promptly using NRT or SC services (Advise) and offered a referral to a free SC service (Refer). Contacts of the participants were sent to the SC service providers of their choice for further treatment (active referral). The above advice was repeated at each follow-up (Do-it-again).

The intervention group additionally received 1-week free NRT-S (Nicotinell; GlaxoSmithKline, Brentford, London, UK) in the original packing (7 NRT patches or 84 pieces of gum). Our previous trial found no difference in quit rates between 1-week or 2-week NRT-S^21^. The dose of the NRT-S was assigned based on the time to the first cigarette of the day^22^. Participants who had their first cigarettes >30 minutes after waking up and had not previously used NRT received 2 mg nicotine gum or 14 mg nicotine patch, while those who smoked ≤30 minutes were given 21 mg nicotine patch (4 mg NRT gum is not available in Hong Kong). The research assistant and trained SC advisors briefly instructed the participants on the usage of NRT and gave an instruction card (Supplementary file Figure 2) containing information on NRT use and potential side effects.

As an extension of the AWARD model at baseline, the intervention group received 12-week personalized behavioral support delivered through SC advisor-delivered IM and a fully automated chatbot. Regular IM messages were guided by the Social Cognitive Theory (SCT)^23^, covering information such as knowledge and skills of quitting, benefits of quitting, strategies to manage urges to smoke for self-efficacy, and SC services, for example, ‘Please identify the important things in your life, which may be related to personal or family health, interpersonal relationships, finances, or others. The important thing can be the driving force for quitting smoking!’. The schedule of messages was adjusted to the participant’s baseline readiness to quit (within the next 7, 30, 60 days or undecided) according to the Transtheoretical Model (TTM)^24^. All participants received a message once a week to initiate an IM conversation. The frequency increased to once daily for the week of the targeted quit date and twice weekly for the week before and after the week of the quit date. The schedule could be adjusted as requested by smokers during IM conversations.

SC advisors interacted in real-time with smokers through IM, providing behavioral support to avoid or handle high-risk situations of smoking (e.g. cigarette invitation from friends), instructions the use of NRT-S and breaking the habitual smoking by time-contingent messages (e.g. first cigarette in the morning). Proactive IM messages such as asking about the recent progress of SC were used to initiate the conversation, for example, ‘During this period of time, I have heard lots of good news one after another. Some people said that they had completely quit smoking, and some had reduced smoking. How about your progress? You can share it with me’. SC advisors delivered SCT- and TTM-guided advice and actively referred the smokers if they expressed the need for SC services.

A rule-based chatbot called ‘Quit Buddy’ had been developed by a multidisciplinary research team comprising experts in public health/ community medicine, computer engineering and experienced SC advisors. See Supplementary file for the structure, schematic of the system, contents, and screenshots of the chatbot. The chatbot was developed for supplementing human SC advisors to answer common queries related to SC, which were identified from the IM dialogues between over 200 smokers and SC advisors in our previous trial^8^. The themes of the questions were quitting methods, craving management, self-efficacy to quit, novel tobacco products, and others, and an input textbox was available for participants to text their queries. Responses to the questions were drafted based on our previous experience in SC counselling and had been further refined according to comments from experienced SC counsellors and service users in Tung Wah Group of Hospitals Integrated Centre on Smoking Cessation, one of the main SC service providers in Hong Kong. Then, a prototype was built using IBM Watson and pilot-tested on 5 smokers recruited from smoking hotspots. The final version of the chatbot was incorporated with Application Programming Interface with a backend server support and continuous data collection. The chatbot was designed as web-based considering that unmotivated smokers were found to be unlikely to download apps for SC^25^. Each participant in the intervention group received a unique link to access the chatbot for tracking individual’s engagement. SC advisors proactively sent a total of 6 reminders of chatbot URL through IM every two weeks during the 12-week personalized behavioral support. The contents of the Chatbot (Supplementary file Table 1) were unchanged during the trial, being accessed only by participants in the intervention group and was not open to the public. The interventions had been archived in a local server. We conducted *post hoc* qualitative interviews with chatbot users after the completion of the trial and reported results elsewhere^26^.

The control group received the same AWARD model as the intervention group at baseline, an established standard care model for Hong Kong community smokers. They additionally received regular SMS messages on healthy lifestyles and reminders to participate in the follow-up surveys for quitting, with a similar frequency to the regular IM sent to the intervention group. Our previous RCT isolating the effect of regular SMS messages on general health resulted in no improvement in quitting^27^.

### Outcomes

Data were collected in person at baseline and through telephone interviews at 3, 6, and 12 months after randomization (intervention initiation). The primary outcomes were carbon monoxide-validated (<4 ppm) smoking abstinence at 6 and 12 months after intervention initiation^28^. Participants who reported having quit tobacco use for 7 days or longer at follow-up at 6 and 12 months were invited for breath carbon monoxide tests. Those who agreed to the tests were given HK$300 (approximately US$38) in cash for their time and travelling expenses.

Secondary outcomes included self-reported 7-day point prevalence and 24-week continuous abstinence; quit attempts; smoking reduction, defined as the self-reported reduction in the number of cigarettes per day by at least 50% of the baseline amount; and SC service use, defined as having attended at least one treatment session delivered by a SC service provider, at 6 and 12 months. Quality of life was assessed at baseline and at 12 months using the validated Chinese EQ-5D-5L questionnaire, with responses transformed using the standard Hong Kong value set form ranging from -0.864, the worst to 1, the best^29^.

### Sample size determination

The sample size was estimated based on our previous trial, which found that the group receiving brief advice and active referral had a 6-month biochemically validated abstinence rate of 9.0% by intention-to-treat analysis^13^. Given an assumed effect size of 1.8 derived from a meta-analysis of mHealth SC RCTs (RR=1.83)^30^, power of 80% and an allocation ratio of 1:1, the required sample size for detecting a significant difference in biochemically validated abstinence rates between the intervention group and control group at two-sided type I error of 0.05 is 664 (each group 332).

### Statistical analysis

All analyses were performed according to a prespecified statistical analysis plan. Primary analyses were by intention-to-treat, assuming participants with missing outcomes to have had no change in smoking behaviors from baseline. Logistic regression was used to compare the SC outcomes between groups. Planned sensitivity analyses were conducted for primary analyses. First, complete case analyses were conducted by excluding participants lost to follow-up. Second, multiple imputation by chained equations assuming data were missing at random was conducted. The imputation models included the outcomes, group allocation, and sociodemographic and baseline smoking-related characteristics that were associated with abstinence or missingness, including sex, age, education level, monthly household income, daily cigarette consumption, time to the first cigarette after waking, previous quit attempt, and readiness to quit^8^. Fifty imputed datasets were generated, and results were pooled according to Rubin’s rule^31^.

We conducted *a priori* subgroup analyses by baseline characteristics, including sex, age group, education level, nicotine dependence level, any previous quit attempt, and readiness to quit in 30 days. Multiplicative interaction terms of baseline characteristics × group allocation were included in logistic regression models to calculate the p values for interaction, although the study was not powered to examine interaction. In the intervention group, we examined the associations of intervention engagement, defined by IM/chatbot use (verified by WhatsApp conversation log^8^ and chatbot backend), self-reported use of NRT-S at 3 months, or both, with validated abstinence outcomes, adjusting for established predictors of SC outcomes, including sex, age, nicotine dependence, previous quit attempt, and readiness to quit^32^. All analyses were conducted in Stata/MP version 15.1. A 2-tailed p<0.05 indicated statistical significance.

## RESULTS

### Participants

**Figure 1:**
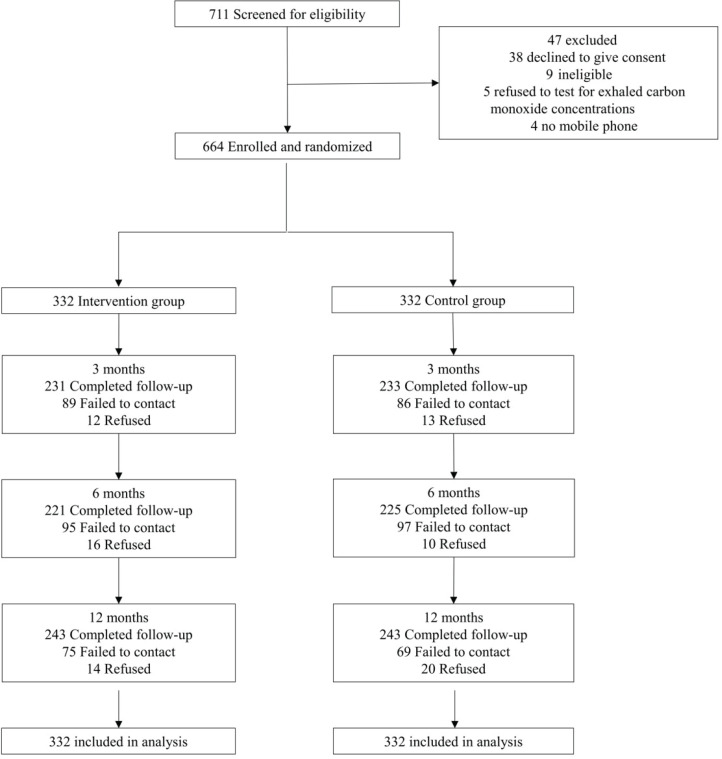
The Consolidated Standards of Reporting Trials (CONSORT) flow diagram

Figure 1 shows that, of 711 smokers screened for eligibility, 664 participants were individually randomized. The retention rate was 69.9%, 67.2%, 73.2% at 3, 6, and 12 months, respectively. Retention rates were similar between the 2 groups (p=0.49–0.95).

Table 1 shows that the baseline characteristics were similar between the 2 groups. Most participants were males (74.4%), aged 18–39 years (63.9%), were single (54.7%), had attained secondary or above education (99.4%), were employed (86.4%), and had monthly household income ≥ HK$30000 (US$1=HK$7.8; 52.7%); 62.4% had low cigarette dependence with Heaviness of Smoking Index 0–2, 59.6% had never made a quit attempt, and 51.7% were not ready to quit in 30 days.

**Table 1:** Baseline characteristics of the participants (N=664)

*Characteristics*	*Intervention (N=332) n (%)^a^*	*Control (N=332) n (%)^a^*	*p ^b^*
**Sex**			0.72
Male	249 (75.0)	245 (73.8)	
Female	83 (25.0)	87 (26.2)	
**Age** (years)			0.92
18–29	99 (30.3)	103 (31.9)	
30–39	104 (31.8)	109 (33.8)	
40–49	78 (23.9)	69 (21.4)	
50–59	35 (10.7)	31 (9.6)	
≥60	11 (3.4)	11 (3.4)	
**Marital status**			0.17
Single	154 (51.2)	175 (58.3)	
Married/cohabited	128 (42.5)	112 (37.3)	
Divorced/separated/widowed	19 (6.3)	13 (4.3)	
**Education level**			0.94
Primary or lower	2 (0.6)	2 (0.6)	
Secondary	160 (48.8)	161 (50.2)	
Tertiary	166 (50.6)	158 (49.2)	
**Employment status**			0.42
Employed	278 (85.0)	282 (87.9)	
Unemployed	41 (12.5)	30 (9.4)	
Retired	8 (2.5)	9 (2.8)	
**Monthly household income** (HK$)^c^			0.99
≤19999	48 (16.5)	48 (16.8)	
20000–29999	90 (30.9)	87 (30.4)	
≥30000	153 (52.6)	151 (52.8)	
**Daily cigarette consumption** (sticks)			0.38
1–10	232 (69.9)	236 (71.1)	
11–20	91 (27.4)	92 (27.7)	
≥21	9 (2.7)	4 (1.2)	
**Time to first cigarette of the day** (minutes)			0.22
>60	97 (29.3)	104 (31.3)	
31–60	57 (17.2)	40 (12.1)	
6–30	89 (26.9)	87 (26.2)	
≤5	88 (26.6)	101 (30.4)	
**Cigarette dependence** (HSI, 0–6)^d^			0.46
Low (0–2)	213 (64.2)	201 (60.5)	
Moderate (3–4)	113 (34.0)	127 (38.3)	
High (5–6)	6 (1.8)	4 (1.2)	
**Previous quit attempt**			0.34
Never	204 (61.5)	192 (57.8)	
Ever	128 (38.6)	140 (42.2)	
**Intention to quit**			0.10
Within next 7 days	84 (25.3)	75 (22.6)	
Within next 30 days	92 (27.7)	70 (21.1)	
Within next 60 days	19 (5.7)	24 (7.2)	
Not decided yet	137 (41.3)	163 (49.1)	
**Perceptions of quitting^e^** (score, 1–10), mean ± SD			
Importance	7.1 ± 2.1	6.8 ± 2.1	0.10
Difficulty	7.3 ± 2.5	7.0 ± 2.4	0.09
Confidence	5.9 ± 2.0	5.7 ± 2.1	0.16

a Sample sizes varied because of missing responses on some variables.

b The p-values were calculated by chi-squared test for categorical variables and t-test for continuous variables.

c US$1= HK$7.8.

d HSI: Heaviness of Smoking Index, a 2-item score from multiple-choice response options (0–3) assessing number of cigarettes smoked per day and latency to smoke after waking, with greater scores indicating higher nicotine dependence.

e Greater scores indicate higher levels of perceptions of quitting.

### Smoking cessation outcomes

Table 2 shows that, by intention-to-treat, the intervention group did not significantly increase biochemically validated abstinence at 6 months (3.9% vs 3.0%; OR=1.31; 95% CI: 0.57–3.04) and 12 months (5.4% vs 4.5%; OR=1.21; 95% CI: 0.60–2.45). Non-significant increases were also shown in selfreported 7-day point-prevalence abstinence, smoking reduction, and use of SC service at 6 and 12 months. The intervention group showed significantly higher rates of quit attempts at 6 months than the control group (47.0% vs 38.0%; OR=1.45; 95% CI: 1.06–1.97). Sensitivity analyses using multiple imputation and complete case analyses yielded similar results (Supplementary file Table 2).

**Table 2:** Primary and secondary outcomes of the participants (N=664)

	*Intervention (N=332)*		*Control (N=332)*		*Regression model*	
	*n*	*%*	*n*	*%*	*OR (95% CI)*	*p*
**Primary outcomes**						
**Validated abstinence**						
6 months	13	3.9	10	3.0	1.31 (0.57–3.04)	0.53
12 months	18	5.4	15	4.5	1.21 (0.60–2.45)	0.59
**Secondary outcomes**						
**Self-reported 7-day point-prevalent abstinence**						
6 months	32	9.6	28	8.4	1.12 (0.68–1.97)	0.59
12 months	34	10.2	32	9.6	1.07 (0.64–1.78)	0.80
**Self-reported 24-week continuous abstinence**						
6 months	14	4.2	19	5.7	0.73 (0.36–1.47)	0.37
12 months	21	6.3	20	6.0	1.05 (0.56–1.98)	0.87
**Smoking reduction by at least 50% of baseline^a^**						
6 months	59/300	19.7	54/304	17.8	1.13 (0.75–1.71)	0.55
12 months	80/298	26.9	67/300	22.3	1.28 (0.88–1.85)	0.20
**Quit attempt**						
6 months (cumulative)	156	47.0	126	38.0	1.45 (1.06–1.97)	0.019
12 months (cumulative)	179	53.9	159	47.9	1.27 (0.94–1.73)	0.12
**Use of smoking cessation service**						
6 months (cumulative)	32	9.6	21	6.3	1.58 (0.89–2.80)	0.12
12 months (cumulative)	42	12.7	33	9.9	1.31 (0.81–2.13)	0.27

a Quitting not included as reduction.

### Subgroup analyses

Table 3 shows that the intervention effect was greater in females (8.4% vs 2.3%; OR=3.91; 95% CI: 0.79–19.42) than in males (4.4% vs 5.3%, OR=0.82; 95% CI: 0.36–1.88) at 12 months and in those who were not ready to quit in 30 days (3.9% vs 1.1%; OR=3.70; 95% CI: 0.74–18.60) than those who were ready to quit in 30 days (4.0% vs 5.5%; OR=0.71; 95% CI: 0.25–2.01) at 6 months with marginal significance of interaction (both p=0.09). Although all interaction effects were not significant (probably due to the small sample size), those who were female, aged 18–29 years, with lower education level (secondary or below), light nicotine dependence, no previous quit attempt, and not ready to quit in 30 days showed greater ORs of quitting at 6 and 12 months.

**Table 3:** Validated abstinence at 6 months and 12 months by subgroups (Intervention, 332; Control, 332)

*Variable*	*6 months*				*12 months*			
	*Intervention n/N (%)*	*Control n/N (%)*	*OR (95% CI)*	*p for interaction*	*Intervention n/N (%)*	*Control n/N (%)*	*OR (95% CI)*	*p for interaction*
**Sex**				0.13				0.09
Male	8/249 (3.2)	9/245 (3.7)	0.87 (0.33–2.29)		11/249 (4.4)	13/245 (5.3)	0.82 (0.36–1.88)	
Female	5/83 (6.0)	1/87 (1.2)	5.51 (0.63–48.22)		7/83 (8.4)	2/87 (2.3)	3.91 (0.79–19.42)	
**Age** (years)				0.46				0.17
18–29	4/99 (4.0)	1/103 (1.0)	4.29 (0.47–39.11)		8/99 (8.1)	3/103 (2.9)	2.93 (0.75–11.38)	
30–39	3/104 (2.9)	3/109 (2.8)	1.05 (0.21–5.32)		4/104 (3.9)	3/109 (2.8)	1.41 (0.31–6.47)	
≥40	6/124 (4.8)	6/111 (5.4)	0.89 (0.28–2.84)		6/124 (4.8)	9/111 (8.1)	0.58 (0.20–1.67)	
**Education level**				0.46				0.49
Secondary or lower	7/162 (4.3)	4/163 (2.5)	1.80 (0.63–6.25)		7/162 (4.3)	4/163 (2.5)	1.80 (0.52–6.25)	
Tertiary	6/166 (3.6)	6/158 (3.8)	0.95 (0.30–3.01)		11/166 (6.6)	10/158 (6.3)	1.05 (0.43–2.55)	
**Nicotine dependence** (Heaviness of smoking Index, 0–6)^b^			0.77				0.17	
Light (0–2)	9/213 (4.2)	6/201 (3.0)	1.43 (0.50–4.10)		16/213 (7.5)	10/201 (5.0)	1.55 (0.69–3.50)	
Moderate to high (3–6)	4/119 (3.4)	4/131 (3.1)	1.10 (0.27–4.52)		2/119 (1.7)	5/131 (3.8)	0.43 (0.08–2.26)	
**Previous quit attempt**				0.63				0.77
Never	7/204 (3.4)	4/192 (2.1)	1.67 (0.48–5.80)		10/204 (4.9)	7/192 (3.7)	1.36 (0.51–3.65)	
Ever	6/128 (4.7)	6/140 (4.3)	1.10 (0.35–3.50)		8/128 (6.3)	8/140 (5.7)	1.10 (0.40–3.02)	
**Readiness to quit in 30 days**				0.09				0.18
No	6/156 (3.9)	2/187 (1.1)	3.70 (0.74–18.60)		6/156 (3.9)	3/187 (1.6)	2.45 (0.60–9.97)	
Yes	7/176 (4.0)	8/145 (5.5)	0.71 (0.25–2.01)		12/176 (6.8)	12/145 (8.3)	0.81 (0.35–1.86)	

a US$1= HK$7.8.

b Heaviness of Smoking Index, a 2-item score from multiple-choice response options (0–3) assessing daily cigarette consumption and time to first cigarette of the day, with greater scores indicating higher nicotine dependence.

### Intervention engagement

In the intervention group, 33.1% (110/332) had used mHealth technologies (IM only: 22.3%, 74/332; chatbot only: 4.0%, 13/332; both IM and chatbot: 7.0%, 23/332) and 25.6% (85/332) had used NRT-S by 3 months. Table 4 shows that, compared with no engagement in IM or chatbot, engagement in IM only showed significantly higher ORs of validated abstinence at 6 months (AOR=4.71; 95% CI: 1.24–17.81) after adjusting for baseline characteristics, and the OR further increased for engagement in both IM and chatbot (AOR=8.95; 95% CI: 1.79–44.75). Of 85 participants who used NRT-S, 67.1% reported no side effect, 11.8% reported headache/dizziness and 8.3% reported skin problems (Supplementary file Table 3). Instructions and support were provided to participants, and no adverse symptoms were reported at follow-up.

**Table 4:** Associations of intervention engagement with validated abstinence at 6 months and 12 months in the intervention group (332 participants)

	*6 months*				*12 months*	
	*Validated abstinence, n/N (%)*	*AOR (95% CI)^a^*	*p*	*Validated abstinence, n/N (%)*	*AOR (95% CI)^a^*	*p*
**Mobile health technologies**						
None (Ref.)	4/222 (1.8)	1		8/222 (3.6)	1	
IM only	6/74 (8.1)	4.71 (1.24–17.81)	0.023	6/74 (8.1)	1.98 (0.64–6.15)	0.24
Chatbot only	0/13 (0)	NA		2/13 (15.4)	4.04 (0.72–22.72)	0.11
Both	3/23 (13.0)	8.95 (1.79–44.75)	0.0076	2/23 (8.7)	2.48 (0.47–12.94)	0.28
**NRT-S**						
No (Ref.)	11/247 (4.5)	1		16/247 (6.5)	1	
Yes	2/85 (2.4)	0.57 (0.12–2.72)	0.49	2/85 (2.4)	0.38 (0.08–1.74)	0.21
**Combined mobile health technologies and NRT-S**						
None (Ref.)	3/175 (1.7)	1		7/175 (4.0)	1	
IM only	5/46 (10.9)	7.09 (1.52–33.17)	0.013	5/46 (10.9)	2.32 (0.65–8.22)	0.19
Chatbot only	0/10 (0)	NA		2/10 (20.0)	4.63 (0.76–28.11)	0.096
Both IM and Chatbot	3/16 (18.8)	13.43 (2.36–76.41)	0.0034	2/16 (12.5)	2.83 (0.52–15.42)	0.23
NRT-S only	1/47 (2.1)	1.49 (0.15–15.21)	0.74	1/47 (2.1)	0.55 (0.06–4.81)	0.59
All	1/38 (2.6)	1.71 (0.17–17.37)	0.65	1/38 (2.6)	0.68 (0.08–5.89)	0.73

IM: instant messaging. NRT-S: nicotine replacement therapy sampling. NA: not applicable.

a AOR: adjusted odds ratio; adjusted for sex, age, nicotine dependence, previous quit attempt, and readiness to quit.

### Quality of life

Participants had only minor problems in their health status at baseline (EQ-5D-5L value: overall: 0.997, intervention: 0.996, control: 0.998) and 12 months (EQ-5D-5L value: overall: 0.998, intervention: 0.997, control: 0.999). No differences were found between the 2 groups.

## DISCUSSION

This pragmatic RCT found that behavioral support through IM and chatbot combined with NRT-S compared with SMS on general health did not significantly improve validated abstinence (primary outcome), self-reported 7-day point-prevalence abstinence, smoking reduction, and use of SC services at 6 and 12 months, in proactively recruited community smokers in Hong Kong. However, engagement with the combined intervention of behavioral support through IM, chatbot, and NRT-S was low in the intervention group.

Our findings were discrepant with the individual and additive effects of mHealth text messaging interventions for SC in the Cochrane review^1^ and meta-analyses^2^. One explanation was the inclusion of many participants who were not motivated to quit (59.6% had never made a quit attempt, and 51.7% were not ready to quit in 30 days). The validated abstinence at 6 months of the intervention group (3.9%) was lower than those of previous RCTs in smokers with high motivation to quit (6.0–11.1%)^3-5^. Note that a direct comparison might not be feasible due to the differences in study settings and populations. Besides, the control group in our group received brief advice and active referral, which has been proven highly effective in increasing quitting in our previous RCT^13^. Through an active referral, the control group was given the same access to conventional, evidence-based smoking cessation treatment (e.g. counselling, NRT, varenicline) as the intervention group. The addition of mobile interventions and NRT-S might provide small additional benefits on quitting, as evidenced by the smaller than expected effect size.

Engagement has been a major challenge for mHealth interventions, particularly for those not ready for behavior change^33^. Similarly, we found low intervention engagement (IM only: 22.3%, 74/332; chatbot only: 4.0%, 13/332; both IM and chatbot: 7.0%, 23/332). Engagement in IM only was associated with a higher abstinence rate, and engagement in both IM and chatbot further increased the abstinence rate at 6 months, suggesting the suboptimal intervention engagement might lead to an underestimation of the intervention effects. Our trial included over 85% of participants were employed, and long working hours, particularly in Hong Kong, might be one of the barriers of IM interactions. Our previous qualitative study showed that ‘too busy’ was one of the most common reasons for not engaging in IM interventions^34^. Future trials on mHealth SC support may balance the busy schedule of participants by extending IM-based service hours. Our qualitative interviews showed that participants in this RCT perceived the chatbot as too robotic and unable to proactively initiate conversation, hence discontinuing the chatbot usage^26^, despite that a total of 6 reminders were proactively sent by SC advisors. Future SC chatbots could incorporate artificial intelligence techniques such as natural language processing and machine learning to better simulate human-to-human interaction. The use of an URL to enter the web-based chatbot might also reduce its convenience and accessibility, which are known barriers of engagement in mHealth interventions^33^. Some SC chatbots have been embedded in popular IM apps to improve the accessibility.

An interesting result was that significantly more quit attempts were shown in the intervention group than the control group at 6 months (47.0% vs 38.0%; OR=1.45; 95% CI: 1.06–1.97). The quit attempt rates, particularly of the intervention group, and effect size were larger than those observed in our previous IM-based interventions in the community (41.0% vs 36.0%; OR=1.27)^8^ and in workplaces (28.6% vs 30.1%; OR=0.95)^35^. Though empirical evidence on the effectiveness of chatbot for SC is limited, a qualitative study showed that chatbot could be used for seeking distraction from cravings and maintaining motivation to quit^36^. Our qualitative findings further indicated that the chatbot could provide more credible information on quitting than other online information sources, and the information acted as quitting reminders for participants^26^. The increase in quit attempts might also be explained by the addition of 1-week NRT-S at baseline, which is known to promote quit attempts for unmotivated smokers^14^. We noted that participants with no previous quit attempts and no readiness to quit in the next 30 days showed stronger intervention effects on validated abstinence at 6 and 12 months, although the interaction effects were non-significant, possibly due to the small sample size. The combination of IM, chatbot, and NRT-S focused on enhancing psychological support and self-efficacy, which might be particularly effective in participants who did not have a motivator to quit. Our findings imply that smokers not having committed to quitting yet might be receptive to mHealth plus NRT-S to make quit attempts or quit smoking.

### Strengths and limitations

Strengths of this trial include an evidence-based, proactive approach to reach community smokers, a randomized study design, long-term follow-up at 6 and 12 months, and biochemical verification of abstinence. Our trial has several limitations. First, the combined interventions restricted us to disentangle the effects of each intervention component (IM, chatbot, NRT-S). IM and NRT-S have been found effective independently in previous SC trials^8,14^. Future RCTs comparing the effects of the interactive chatbot with one-way text messaging are warranted for advancing mHealth strategies for SC. Second, at 6 and 12 months, 32.8% and 26.8% of participants were lost to follow-up, respectively, and 61.7% and 50.0% of self-reported abstinence were not biochemically verified, comparable with our previous low-contact mobile SC RCT^8^. Nevertheless, our sensitivity analyses using multiple imputations and by complete case yielded similar results by intention-to-treat, reducing concerns about non-response bias. Third, the effects of interventions on SC might be influenced by unmeasured factors, such as the duration participants had been smoking and digital health literacy. Fourth, participants were mainly male and were mostly with a low to moderate level of nicotine dependence, without past quit attempts, and not ready to quit in the short-term. The generalizability of our results is uncertain to other populations with different sociodemographic and smoking characteristics. Fifth, this trial may be less applicable to other interventions that do not refer to SC services or in other settings with limited SC services. Sixth, future user-centered research may explore the usability, easiness of use, and aesthetic design of the chatbot.

## CONCLUSIONS

Mobile interventions plus NRT-S showed small non-significant increases in abstinence in community smokers. Improving intervention engagement is needed to maximize the effectiveness of such interventions.

## Data Availability

The data supporting this research are available from the authors on reasonable request.
