# Supplementary material for: Effect of mobile interventions with nicotine replacement therapy sampling on long-term smoking cessation in community smokers: A pragmatic randomized clinical trial
Source: Tob Induc Dis. 2023 Mar 24;21:44. doi: 10.18332/tid/160168 (PMC10037427; doi:10.18332/tid/160168)
Supplement: Supplementary file 1 [file TID-21-44-s1.pdf]

CONSORT-EHEALTH (V 1.6.1) - Submission/Publication Form

The CONSORT-EHEALTH checklist is intended for authors of randomized trials evaluating web-based and Internet-based applications/interventions, including mobile interventions, electronic games (incl multiplayer games), social media, certain telehealth applications, and other interactive and/or networked electronic applications. Some of the items (e.g. all subitems under item 5 - description of the intervention) may also be applicable for other study designs.

The goal of the CONSORT EHEALTH checklist and guideline is to be
a) a guide for reporting for authors of RCTs,
b) to form a basis for appraisal of an ehealth trial (in terms of validity)

CONSORT-EHEALTH items/subitems are MANDATORY reporting items for studies published in the Journal of Medical Internet Research and other journals / scientific societies endorsing the checklist.

Items numbered 1., 2., 3., 4a., 4b etc are original CONSORT or CONSORT-NPT (non-pharmacologic treatment) items.
Items with Roman numerals (i., ii, iii, iv etc.) are CONSORT-EHEALTH extensions/clarifications.

As the CONSORT-EHEALTH checklist is still considered in a formative stage, we would ask that you also RATE ON A SCALE OF 1-5 how important/useful you feel each item is FOR THE PURPOSE OF THE CHECKLIST and reporting guideline (optional).

Mandatory reporting items are marked with a red *.
In the textboxes, either copy & paste the relevant sections from your manuscript into this form - please include any quotes from your manuscript in QUOTATION MARKS,
or answer directly by providing additional information not in the manuscript, or elaborating on why the item was not relevant for this study.

YOUR ANSWERS WILL BE PUBLISHED AS A SUPPLEMENTARY FILE TO YOUR PUBLICATION IN JMIR AND ARE CONSIDERED PART OF YOUR PUBLICATION (IF ACCEPTED).
Please fill in these questions diligently. Information will not be copyedited, so please use proper spelling and grammar, use correct capitalization, and avoid abbreviations.

DO NOT FORGET TO SAVE AS PDF _AND_ CLICK THE SUBMIT BUTTON SO YOUR ANSWERS ARE IN OUR DATABASE !!!

Citation Suggestion (if you append the pdf as Appendix we suggest to cite this paper in the caption):
Eysenbach G, CONSORT-EHEALTH Group
CONSORT-EHEALTH: Improving and Standardizing Evaluation Reports of Web-based and Mobile Health Interventions
J Med Internet Res 2011;13(4):e126
URL: http://www.jmir.org/2011/4/e126/
doi: 10.2196/jmir.1923
PMID: 22209829

nyguo@connect.hku.hk (not shared) Switch account   Draft not saved

* Required

Your name *
First Last

Ningyuan Guo

Primary Affiliation (short), City, Country *

University of Toronto, Toronto, Canada

Shanghai Jiao Tong University, Shanghai, Chin[illegible]

Your e-mail address *

abc@gmail.com

nyguo@shsmu.edu.cn

Title of your manuscript *

Provide the (draft) title of your manuscript.

Effect of mobile health technologies and nicotine replacement therapy sampling on long-term smoking cessation in community smokers: a pragmatic randomized clinical trial

Name of your App/Software/Intervention *

If there is a short and a long/alternate name, write the short name first and add the long name in brackets.

IM and chatbot (Quit Buddy)

Evaluated Version (if any)

e.g. "V1", "Release 2017-03-01", "Version 2.0.27913"

Your answer

Language(s) *

What language is the intervention/app in? If multiple languages are available, separate by comma (e.g. "English, French")

English

URL of your Intervention Website or App

e.g. a direct link to the mobile app on app in appstore (itunes, Google Play), or URL of the website. If the intervention is a DVD or hardware, you can also link to an Amazon page.

https://quitbuddy-bot.hkuteli.net/api/web/users

URL of an image/screenshot (optional)

Your answer

Accessibility *

Can an enduser access the intervention presently?

- ◯ access is free and open
- ◉ access only for special usergroups, not open
- ◯ access is open to everyone, but requires payment/subscription/in-app purchases
- ◯ app/intervention no longer accessible
- ◯ Other: ____________

Primary Medical Indication/Disease/Condition *

e.g. "Stress", "Diabetes", or define the target group in brackets after the condition, e.g. "Autism (Parents of children with)", "Alzheimers (Informal Caregivers of)"

Tobacco dependence

Primary Outcomes measured in trial *

comma-separated list of primary outcomes reported in the trial

Carbon monoxide-validated smoking abstinen[illegible]

Secondary/other outcomes

Are there any other outcomes the intervention is expected to affect?

Self-reported 7-day point-prevalence and 24-week continuous abstinence, quit attempts, smoking reduction, and SC service use at 6 and 12 months

Recommended "Dose" *

What do the instructions for users say on how often the app should be used?

- ◯ Approximately Daily
- ◯ Approximately Weekly
- ◯ Approximately Monthly
- ◯ Approximately Yearly
- ◉ "as needed"
- ◯ Other: ____________

Approx. Percentage of Users (starters) still using the app as recommended after 3 months *

- ( ) unknown / not evaluated
- ( ) 0-10%
- (•) 11-20%
- ( ) 21-30%
- ( ) 31-40%
- ( ) 41-50%
- ( ) 51-60%
- ( ) 61-70%
- ( ) 71%-80%
- ( ) 81-90%
- ( ) 91-100%
- ( ) Other: ____________

Overall, was the app/intervention effective? *

- ( ) yes: all primary outcomes were significantly better in intervention group vs control
- ( ) partly: SOME primary outcomes were significantly better in intervention group vs control
- (•) no statistically significant difference between control and intervention
- ( ) potentially harmful: control was significantly better than intervention in one or more outcomes
- ( ) inconclusive: more research is needed
- ( ) Other: ____________

Article Preparation Status/Stage *

At which stage in your article preparation are you currently (at the time you fill in this form)

- ( ) not submitted yet - in early draft status
- ( ) not submitted yet - in late draft status, just before submission
- ( ) submitted to a journal but not reviewed yet
- (•) submitted to a journal and after receiving initial reviewer comments
- ( ) submitted to a journal and accepted, but not published yet
- ( ) published
- ( ) Other: ____________

Journal *

If you already know where you will submit this paper (or if it is already submitted), please provide the journal name (if it is not JMIR, provide the journal name under "other")

- ( ) not submitted yet / unclear where I will submit this
- ( ) Journal of Medical Internet Research (JMIR)
- ( ) JMIR mHealth and UHealth
- ( ) JMIR Serious Games
- ( ) JMIR Mental Health
- ( ) JMIR Public Health
- ( ) JMIR Formative Research
- ( ) Other JMIR sister journal
- (x) Other: Tobacco Induced Diseases

Is this a full powered effectiveness trial or a pilot/feasibility trial? *

- ( ) Pilot/feasibility
- (x) Fully powered

Manuscript tracking number *

If this is a JMIR submission, please provide the manuscript tracking number under "other" (The ms tracking number can be found in the submission acknowledgement email, or when you login as author in JMIR. If the paper is already published in JMIR, then the ms tracking number is the four-digit number at the end of the DOI, to be found at the bottom of each published article in JMIR)

- ( ) no ms number (yet) / not (yet) submitted to / published in JMIR
- (x) Other: The paper is not a JMIR submission.

TITLE AND ABSTRACT

1a) TITLE: Identification as a randomized trial in the title

1a) Does your paper address CONSORT item 1a? *

I.e does the title contain the phrase "Randomized Controlled Trial"? (if not, explain the reason under "other")

- (x) yes
- ( ) Other:

1a-i) Identify the mode of delivery in the title

Identify the mode of delivery. Preferably use “web-based” and/or “mobile” and/or “electronic game” in the title. Avoid ambiguous terms like “online”, “virtual”, “interactive”. Use “Internet-based” only if Intervention includes non-web-based Internet components (e.g. email), use “computer-based” or “electronic” only if offline products are used. Use “virtual” only in the context of “virtual reality” (3-D worlds). Use “online” only in the context of “online support groups”. Complement or substitute product names with broader terms for the class of products (such as “mobile” or “smart phone” instead of “iphone”), especially if the application runs on different platforms.

	1	2	3	4	5	
subitem not at all important	○	○	○	○	◉	essential

Clear selection

Does your paper address subitem 1a-i? *

Copy and paste relevant sections from manuscript title (include quotes in quotation marks "like this" to indicate direct quotes from your manuscript), or elaborate on this item by providing additional information not in the ms, or briefly explain why the item is not applicable/relevant for your study

mobile health technologies

1a-ii) Non-web-based components or important co-interventions in title

Mention non-web-based components or important co-interventions in title, if any (e.g., “with telephone support”).

	1	2	3	4	5	
subitem not at all important	○	○	○	○	◉	essential

Clear selection

Does your paper address subitem 1a-ii?

Copy and paste relevant sections from manuscript title (include quotes in quotation marks "like this" to indicate direct quotes from your manuscript), or elaborate on this item by providing additional information not in the ms, or briefly explain why the item is not applicable/relevant for your study

nicotine replacement therapy sampling

1a-iii) Primary condition or target group in the title

Mention primary condition or target group in the title, if any (e.g., “for children with Type I Diabetes”) Example: A Web-based and Mobile Intervention with Telephone Support for Children with Type I Diabetes: Randomized Controlled Trial

	1	2	3	4	5	
subitem not at all important	○	○	○	○	◉	essential

Clear selection

Does your paper address subitem 1a-iii? *

Copy and paste relevant sections from manuscript title (include quotes in quotation marks "like this" to indicate direct quotes from your manuscript), or elaborate on this item by providing additional information not in the ms, or briefly explain why the item is not applicable/relevant for your study

long-term smoking cessation in community smokers

1b) ABSTRACT: Structured summary of trial design, methods, results, and conclusions

NPT extension: Description of experimental treatment, comparator, care providers, centers, and blinding status.

1b-i) Key features/functionalities/components of the intervention and comparator in the METHODS section of the ABSTRACT

Mention key features/functionalities/components of the intervention and comparator in the abstract. If possible, also mention theories and principles used for designing the site. Keep in mind the needs of systematic reviewers and indexers by including important synonyms. (Note: Only report in the abstract what the main paper is reporting. If this information is missing from the main body of text, consider adding it)

	1	2	3	4	5	
subitem not at all important	○	○	○	○	◉	essential

Clear selection

Does your paper address subitem 1b-i? *

Copy and paste relevant sections from the manuscript abstract (include quotes in quotation marks "like this" to indicate direct quotes from your manuscript), or elaborate on this item by providing additional information not in the ms, or briefly explain why the item is not applicable/relevant for your study

Both groups received brief advice and active referral to SC services. The intervention group received 1-week NRT-S at baseline and 12-week behavioral support through SC advisor-delivered IM and a fully automated chatbot. The control group received regular text messages regarding general health at a similar frequency.

1b-ii) Level of human involvement in the METHODS section of the ABSTRACT

Clarify the level of human involvement in the abstract, e.g., use phrases like “fully automated” vs. “therapist/nurse/care provider/physician-assisted” (mention number and expertise of providers involved, if any). (Note: Only report in the abstract what the main paper is reporting. If this information is missing from the main body of text, consider adding it)

	1	2	3	4	5	
subitem not at all important	○	○	○	○	◉	essential

Clear selection

Does your paper address subitem 1b-ii?

Copy and paste relevant sections from the manuscript abstract (include quotes in quotation marks "like this" to indicate direct quotes from your manuscript), or elaborate on this item by providing additional information not in the ms, or briefly explain why the item is not applicable/relevant for your study

The intervention group received 1-week NRT-S at baseline and 12-week behavioral support through SC advisor-delivered IM and a fully automated chatbot.

1b-iii) Open vs. closed, web-based (self-assessment) vs. face-to-face assessments in the METHODS section of the ABSTRACT

Mention how participants were recruited (online vs. offline), e.g., from an open access website or from a clinic or a closed online user group (closed usergroup trial), and clarify if this was a purely web-based trial, or there were face-to-face components (as part of the intervention or for assessment). Clearly say if outcomes were self-assessed through questionnaires (as common in web-based trials). Note: In traditional offline trials, an open trial (open-label trial) is a type of clinical trial in which both the researchers and participants know which treatment is being administered. To avoid confusion, use “blinded” or “unblinded” to indicated the level of blinding instead of “open”, as “open” in web-based trials usually refers to “open access” (i.e. participants can self-enrol). (Note: Only report in the abstract what the main paper is reporting. If this information is missing from the main body of text, consider adding it)

	1	2	3	4	5	
subitem not at all important	○	○	○	○	◉	essential

Clear selection

Does your paper address subitem 1b-iii?

Copy and paste relevant sections from the manuscript abstract (include quotes in quotation marks "like this" to indicate direct quotes from your manuscript), or elaborate on this item by providing additional information not in the ms, or briefly explain why the item is not applicable/relevant for your study

664 adult daily cigarette smokers (74.4% male, 51.7% not ready to quit in 30 days) were proactively recruited from smoking hotspots

1b-iv) RESULTS section in abstract must contain use data

Report number of participants enrolled/assessed in each group, the use/uptake of the intervention (e.g., attrition/adherence metrics, use over time, number of logins etc.), in addition to primary/secondary outcomes. (Note: Only report in the abstract what the main paper is reporting. If this information is missing from the main body of text, consider adding it)

	1	2	3	4	5	
subitem not at all important	○	○	○	○	◉	essential

Clear selection

Does your paper address subitem 1b-iv?

Copy and paste relevant sections from the manuscript abstract (include quotes in quotation marks "like this" to indicate direct quotes from your manuscript), or elaborate on this item by providing additional information not in the ms, or briefly explain why the item is not applicable/relevant for your study

Intervention engagement rates were low (IM only: 22.3%; chatbot only: 4.0%; both: 7.0%), but engagement in IM alone or combined with chatbot showed higher abstinence at 6 months (adjusted ORs=4.71 and 8.95, both P<0.05).

1b-v) CONCLUSIONS/DISCUSSION in abstract for negative trials

Conclusions/Discussions in abstract for negative trials: Discuss the primary outcome - if the trial is negative (primary outcome not changed), and the intervention was not used, discuss whether negative results are attributable to lack of uptake and discuss reasons. (Note: Only report in the abstract what the main paper is reporting. If this information is missing from the main body of text, consider adding it)

	1	2	3	4	5	
subitem not at all important	○	○	○	○	◉	essential

Clear selection

Does your paper address subitem 1b-v?

Copy and paste relevant sections from the manuscript abstract (include quotes in quotation marks "like this" to indicate direct quotes from your manuscript), or elaborate on this item by providing additional information not in the ms, or briefly explain why the item is not applicable/relevant for your study

mHealth technologies plus NRT-S did not significantly improve abstinence in community smokers. The low intervention engagement needed to be addressed in future studies.

INTRODUCTION

2a) In INTRODUCTION: Scientific background and explanation of rationale

2a-i) Problem and the type of system/solution

Describe the problem and the type of system/solution that is object of the study: intended as stand-alone intervention vs. incorporated in broader health care program? Intended for a particular patient population? Goals of the intervention, e.g., being more cost-effective to other interventions, replace or complement other solutions? (Note: Details about the intervention are provided in “Methods” under 5)

	1	2	3	4	5	
subitem not at all important	○	○	○	○	◉	essential

Clear selection

Does your paper address subitem 2a-i? *

Copy and paste relevant sections from the manuscript (include quotes in quotation marks "like this" to indicate direct quotes from your manuscript), or elaborate on this item by providing additional information not in the ms, or briefly explain why the item is not applicable/relevant for your study

Longer term effect of mHealth SC interventions is uncertain as few RCTs (15%) had a follow up beyond 6 months.
The population-level effect of mHealth interventions remains unknown as many who do not want to quit or plan to quit were not included.
mHealth interventions in the community smokers with longer follow-up length are needed.
The present RCT developed a chatbot in addition to the established AWARD model, IM-based intervention, and NRT-S. We aimed to evaluate the long-term (6 and 12 months) effect of such a combined intervention on smoking abstinence in Hong Kong community smokers.

2a-ii) Scientific background, rationale: What is known about the (type of) system

Scientific background, rationale: What is known about the (type of) system that is the object of the study (be sure to discuss the use of similar systems for other conditions/diagnoses, if appropiate), motivation for the study, i.e. what are the reasons for and what is the context for this specific study, from which stakeholder viewpoint is the study performed, potential impact of findings [2]. Briefly justify the choice of the comparator.

	1	2	3	4	5	
subitem not at all important	○	○	○	○	◉	essential

Clear selection

Does your paper address subitem 2a-ii? *

Copy and paste relevant sections from the manuscript (include quotes in quotation marks "like this" to indicate direct quotes from your manuscript), or elaborate on this item by providing additional information not in the ms, or briefly explain why the item is not applicable/relevant for your study

Instant messaging (IM, e.g., WhatsApp, WeChat) is a popular and inexpensive alternative to SMS. Our qualitative interviews on community smokers (76% had no quit plan in the next 6 months) showed that the provision of more personalized behavioral support from human SC advisors was the most valued utility of IM for SC.8 Our pragmatic RCT further showed that IM intervention was effective for SC in community smokers.9 However, the intervention engagement rate was low (17%), which might be due to the unavailability of human SC advisors outside office hours.9 SC support could be sustained using chatbots (also known as conversational agents), online computer programs that can simulate human conversations. Evidence on chatbots for SC is emerging but remains scarce and limited. A formative study showed that a chatbot increased motivation to quit immediately after usage in a volunteer sample of young smokers.10 An RCT focusing on smokers motivated to quit identified that adding a chatbot to an SC app more than doubled intervention engagement with the app (incidence rate ratios=2.01, 95% CI 1.92, 2.11), but the effect on SC was unclear because of a low retention rate at 1 month (10.7%).11 A pragmatic RCT in primary care settings showed that a chatbot was marginally more effective than usual care (biochemically validated abstinence at 6 months: odds ratio[OR]=1.52, 95% CI 1.00, 2.31; P=0.05) despite of potential non-response bias due to a low retention rate (45.2%).12
The interventions in the present RCT were developed based on established evidence of our prior studies and RCTs. We have developed and tested an approach13 of proactively reaching community smokers who were largely unmotivated to quit and reasonably representative of the general smoking population regarding their sociodemographic and smoking characteristics.9,14 Our 2015 RCT in the proactively recruited community smokers showed that brief advice using the AWARD model (Ask, Warning, Advice, Referral, Do-it-again) was effective for SC.15 Our 2017 RCT further developed the IM-based intervention combined with the AWARD model and supported the effectiveness for SC.9 Nicotine replacement therapy sampling (NRT-S) has been used in unmotivated smokers and was found to be effective for increasing quit attempts and full-course NRT use.16 A recent RCT in unmotivated smokers showed that mHealth intervention plus NRT-S led to higher abstinence at 6 months than NRT-S alone.17 Our pilot RCT showed that the IM-based intervention plus NRT-S was feasible with positive effects on quitting, smoking reduction, quit attempts, and NRT-S use in community smokers.18

2b) In INTRODUCTION: Specific objectives or hypotheses

Does your paper address CONSORT subitem 2b? *

Copy and paste relevant sections from the manuscript (include quotes in quotation marks "like this" to indicate direct quotes from your manuscript), or elaborate on this item by providing additional information not in the ms, or briefly explain why the item is not applicable/relevant for your study

The present RCT developed a chatbot in addition to the established AWARD model, IM-based intervention, and NRT-S. We aimed to evaluate the long-term (6 and 12 months) effect of such a combined intervention on smoking abstinence in Hong Kong community smokers.

METHODS

3a) Description of trial design (such as parallel, factorial) including allocation ratio

Does your paper address CONSORT subitem 3a? *

Copy and paste relevant sections from the manuscript (include quotes in quotation marks "like this" to indicate direct quotes from your manuscript), or elaborate on this item by providing additional information not in the ms, or briefly explain why the item is not applicable/relevant for your study

This was a two-arm, parallel, assessor-blinded randomized controlled trial;
The randomization sequence with a 1:1 allocation ratio and permuted block of 4, 8, or 12 was generated by a non-investigator.

3b) Important changes to methods after trial commencement (such as eligibility criteria), with reasons

Does your paper address CONSORT subitem 3b? *

Copy and paste relevant sections from the manuscript (include quotes in quotation marks "like this" to indicate direct quotes from your manuscript), or elaborate on this item by providing additional information not in the ms, or briefly explain why the item is not applicable/relevant for your study

No changes to methods after trial commencement

3b-i) Bug fixes, Downtimes, Content Changes

Bug fixes, Downtimes, Content Changes: ehealth systems are often dynamic systems. A description of changes to methods therefore also includes important changes made on the intervention or comparator during the trial (e.g., major bug fixes or changes in the functionality or content) (5-iii) and other “unexpected events” that may have influenced study design such as staff changes, system failures/downtimes, etc. [2].

	1	2	3	4	5	
subitem not at all important	○	○	○	●	○	essential

Clear selection

Does your paper address subitem 3b-i?

Copy and paste relevant sections from the manuscript (include quotes in quotation marks "like this" to indicate direct quotes from your manuscript), or elaborate on this item by providing additional information not in the ms, or briefly explain why the item is not applicable/relevant for your study

No Bug fixes, Downtimes, Content Changes

4a) Eligibility criteria for participants

Does your paper address CONSORT subitem 4a? *

Copy and paste relevant sections from the manuscript (include quotes in quotation marks "like this" to indicate direct quotes from your manuscript), or elaborate on this item by providing additional information not in the ms, or briefly explain why the item is not applicable/relevant for your study

We recruited Hong Kong resident aged ≥18 years who was able to read and communicate in Chinese; currently smoked at least one cigarette daily, validated by an exhaled carbon monoxide level of ≥4 parts per million (ppm); and owned a smartphone and were willing to install an IM app (if not already installed).

4a-i) Computer / Internet literacy

Computer / Internet literacy is often an implicit "de facto" eligibility criterion - this should be explicitly clarified.

	1	2	3	4	5	
subitem not at all important	○	○	○	◉	○	essential

Clear selection

Does your paper address subitem 4a-i?

Copy and paste relevant sections from the manuscript (include quotes in quotation marks "like this" to indicate direct quotes from your manuscript), or elaborate on this item by providing additional information not in the ms, or briefly explain why the item is not applicable/relevant for your study

Computer/internet literacy was not measured and has been acknowledged as a limitation.

4a-ii) Open vs. closed, web-based vs. face-to-face assessments:

Open vs. closed, web-based vs. face-to-face assessments: Mention how participants were recruited (online vs. offline), e.g., from an open access website or from a clinic, and clarify if this was a purely web-based trial, or there were face-to-face components (as part of the intervention or for assessment), i.e., to what degree got the study team to know the participant. In online-only trials, clarify if participants were quasi-anonymous and whether having multiple identities was possible or whether technical or logistical measures (e.g., cookies, email confirmation, phone calls) were used to detect/prevent these.

	1	2	3	4	5	
subitem not at all important	○	○	○	○	◉	essential

Clear selection

Does your paper address subitem 4a-ii? *

Copy and paste relevant sections from the manuscript (include quotes in quotation marks "like this" to indicate direct quotes from your manuscript), or elaborate on this item by providing additional information not in the ms, or briefly explain why the item is not applicable/relevant for your study

Participants were proactively recruited from smoking hotspots, outdoor places where smokers gather and smoke (e.g. exits of underground transit and railway stations, shopping malls, and large commercial buildings), throughout Hong Kong from August 19, 2019 to May 8, 2020.

4a-iii) Information giving during recruitment

Information given during recruitment. Specify how participants were briefed for recruitment and in the informed consent procedures (e.g., publish the informed consent documentation as appendix, see also item X26), as this information may have an effect on user self-selection, user expectation and may also bias results.

	1	2	3	4	5	
subitem not at all important	○	○	○	○	●	essential

Clear selection

Does your paper address subitem 4a-iii?

Copy and paste relevant sections from the manuscript (include quotes in quotation marks "like this" to indicate direct quotes from your manuscript), or elaborate on this item by providing additional information not in the ms, or briefly explain why the item is not applicable/relevant for your study

Smokers were asked about smoking behaviors, assessed exhaled carbon monoxide level, and invited to participate in the study. Those showing interests were assessed for eligibility, and written informed consents were sought.

4b) Settings and locations where the data were collected

Does your paper address CONSORT subitem 4b? *

Copy and paste relevant sections from the manuscript (include quotes in quotation marks "like this" to indicate direct quotes from your manuscript), or elaborate on this item by providing additional information not in the ms, or briefly explain why the item is not applicable/relevant for your study

Eligible participants completed a brief self-administered baseline questionnaire to provide data on sociodemographic and smoking characteristics and quality of life. To avoid intervention contamination, 1 smoker was randomly approached when there were more smokers at the same hotspot. Smoking-related outcomes were measured in follow-up questionnaires through telephone interviews at 3, 6, and 12 months after randomization (intervention initiation), and quality of life was additionally assessed at 12 months.

4b-i) Report if outcomes were (self-)assessed through online questionnaires

Clearly report if outcomes were (self-)assessed through online questionnaires (as common in web-based trials) or otherwise.

	1	2	3	4	5	
subitem not at all important	○	○	○	○	●	essential

Clear selection

Does your paper address subitem 4b-i? *

Copy and paste relevant sections from the manuscript (include quotes in quotation marks "like this" to indicate direct quotes from your manuscript), or elaborate on this item by providing additional information not in the ms, or briefly explain why the item is not applicable/relevant for your study

Smoking-related outcomes were measured in follow-up questionnaires through telephone interviews at 3, 6, and 12 months after randomization (intervention initiation), and quality of life was additionally assessed at 12 months.

4b-ii) Report how institutional affiliations are displayed

Report how institutional affiliations are displayed to potential participants [on ehealth media], as affiliations with prestigious hospitals or universities may affect volunteer rates, use, and reactions with regards to an intervention.(Not a required item – describe only if this may bias results)

	1	2	3	4	5	
subitem not at all important	○	○	○	○	○	essential

Does your paper address subitem 4b-ii?

Copy and paste relevant sections from the manuscript (include quotes in quotation marks "like this" to indicate direct quotes from your manuscript), or elaborate on this item by providing additional information not in the ms, or briefly explain why the item is not applicable/relevant for your study

Your answer

5) The interventions for each group with sufficient details to allow replication, including how and when they were actually administered

5-i) Mention names, credential, affiliations of the developers, sponsors, and owners

Mention names, credential, affiliations of the developers, sponsors, and owners [6] (if authors/evaluators are owners or developer of the software, this needs to be declared in a "Conflict of interest" section or mentioned elsewhere in the manuscript).

	1	2	3	4	5	
subitem not at all important	○	○	◉	○	○	essential

Clear selection

Does your paper address subitem 5-i?

Copy and paste relevant sections from the manuscript (include quotes in quotation marks "like this" to indicate direct quotes from your manuscript), or elaborate on this item by providing additional information not in the ms, or briefly explain why the item is not applicable/relevant for your study

Interventions section: A rule-based chatbot called "Quit Buddy" had been developed by a multidisciplinary research team comprising experts in public health/ community medicine, computer engineering and experienced SC advisors.

5-ii) Describe the history/development process

Describe the history/development process of the application and previous formative evaluations (e.g., focus groups, usability testing), as these will have an impact on adoption/use rates and help with interpreting results.

	1	2	3	4	5	
subitem not at all important	○	○	○	○	◉	essential

Clear selection

Does your paper address subitem 5-ii?

Copy and paste relevant sections from the manuscript (include quotes in quotation marks "like this" to indicate direct quotes from your manuscript), or elaborate on this item by providing additional information not in the ms, or briefly explain why the item is not applicable/relevant for your study

Regular IM messages were guided by the Social Cognitive Theory (SCT), covering information such as knowledge and skills of quitting, benefits of quitting, strategies to manage urges to smoke for self-efficacy, and SC services, for example, "Please identify the important things in your life, which may be related to personal or family health, interpersonal relationships, finances, or others. The important thing can be the driving force for quitting smoking!" The schedule of messages was adjusted to the participants' baseline readiness to quit (within next 7 days/ 30 days/ 60 days or undecided) as according to the Transtheoretical Model (TTM).

Responses to the questions were drafted based on our previous experience in SC counseling and had been further refined according to comments from experienced SC counselors and service users in Tung Wah Group of Hospitals Integrated Centre on Smoking Cessation, one of the main SC service providers in Hong Kong. Then, a prototype had been built using IBM Watson and pilot-tested in 5 smokers recruited from smoking hotspots. The final version of Chatbot was incorporated with Application Programming Interface with a backend server support and continuous data collection.

5-iii) Revisions and updating

Revisions and updating. Clearly mention the date and/or version number of the application/intervention (and comparator, if applicable) evaluated, or describe whether the intervention underwent major changes during the evaluation process, or whether the development and/or content was "frozen" during the trial. Describe dynamic components such as news feeds or changing content which may have an impact on the replicability of the intervention (for unexpected events see item 3b).

	1	2	3	4	5	
subitem not at all important	○	○	◉	○	○	essential

Clear selection

Does your paper address subitem 5-iii?

Copy and paste relevant sections from the manuscript (include quotes in quotation marks "like this" to indicate direct quotes from your manuscript), or elaborate on this item by providing additional information not in the ms, or briefly explain why the item is not applicable/relevant for your study

Contents of the Chatbot were unchanged during the trial.

5-iv) Quality assurance methods

Provide information on quality assurance methods to ensure accuracy and quality of information provided [1], if applicable.

	1	2	3	4	5	
subitem not at all important	○	○	○	◉	○	essential

Clear selection

Does your paper address subitem 5-iv?

Copy and paste relevant sections from the manuscript (include quotes in quotation marks "like this" to indicate direct quotes from your manuscript), or elaborate on this item by providing additional information not in the ms, or briefly explain why the item is not applicable/relevant for your study

The final version of Chatbot was incorporated with Application Programming Interface with a backend server support and continuous data collection... Each participant in the intervention group received a unique link to access the chatbot for tracking individual's engagement.

5-v) Ensure replicability by publishing the source code, and/or providing screenshots/screen-capture video, and/or providing flowcharts of the algorithms used

Ensure replicability by publishing the source code, and/or providing screenshots/screen-capture video, and/or providing flowcharts of the algorithms used. Replicability (i.e., other researchers should in principle be able to replicate the study) is a hallmark of scientific reporting.

	1	2	3	4	5	
subitem not at all important	○	○	○	○	◉	essential

Clear selection

Does your paper address subitem 5-v?

Copy and paste relevant sections from the manuscript (include quotes in quotation marks "like this" to indicate direct quotes from your manuscript), or elaborate on this item by providing additional information not in the ms, or briefly explain why the item is not applicable/relevant for your study

Please see Supplement file 3.

5-vi) Digital preservation

Digital preservation: Provide the URL of the application, but as the intervention is likely to change or disappear over the course of the years; also make sure the intervention is archived (Internet Archive, webcitation.org, and/or publishing the source code or screenshots/videos alongside the article). As pages behind login screens cannot be archived, consider creating demo pages which are accessible without login.

	1	2	3	4	5	
subitem not at all important	○	○	◉	○	○	essential

Clear selection

Does your paper address subitem 5-vi?

Copy and paste relevant sections from the manuscript (include quotes in quotation marks "like this" to indicate direct quotes from your manuscript), or elaborate on this item by providing additional information not in the ms, or briefly explain why the item is not applicable/relevant for your study

URL is presented as follows: https://quitbuddy-bot.hkuteli.net/api/web/users;
The interventions had been archived in local server.

5-vii) Access

Access: Describe how participants accessed the application, in what setting/context, if they had to pay (or were paid) or not, whether they had to be a member of specific group. If known, describe how participants obtained “access to the platform and Internet” [1]. To ensure access for editors/reviewers/readers, consider to provide a “backdoor” login account or demo mode for reviewers/readers to explore the application (also important for archiving purposes, see vi).

	1	2	3	4	5	
subitem not at all important	○	○	○	○	◉	essential

Clear selection

Does your paper address subitem 5-vii? *

Copy and paste relevant sections from the manuscript (include quotes in quotation marks "like this" to indicate direct quotes from your manuscript), or elaborate on this item by providing additional information not in the ms, or briefly explain why the item is not applicable/relevant for your study

The chatbot was designed as web-based considering that unmotivated smokers were found to be unlikely to download apps for SC. Each participant in the intervention group received a unique link to access the chatbot for tracking individual’s engagement.

5-viii) Mode of delivery, features/functionalities/components of the intervention and comparator, and the theoretical framework

Describe mode of delivery, features/functionalities/components of the intervention and comparator, and the theoretical framework [6] used to design them (instructional strategy [1], behaviour change techniques, persuasive features, etc., see e.g., [7, 8] for terminology). This includes an in-depth description of the content (including where it is coming from and who developed it) [1],” whether [and how] it is tailored to individual circumstances and allows users to track their progress and receive feedback” [6]. This also includes a description of communication delivery channels and – if computer-mediated communication is a component – whether communication was synchronous or asynchronous [6]. It also includes information on presentation strategies [1], including page design principles, average amount of text on pages, presence of hyperlinks to other resources, etc. [1].

	1	2	3	4	5	
subitem not at all important	○	○	○	○	◉	essential

Clear selection

Does your paper address subitem 5-viii? *

Copy and paste relevant sections from the manuscript (include quotes in quotation marks "like this" to indicate direct quotes from your manuscript), or elaborate on this item by providing additional information not in the ms, or briefly explain why the item is not applicable/relevant for your study

Regular IM messages were guided by the Social Cognitive Theory (SCT), covering information such as knowledge and skills of quitting, benefits of quitting, strategies to manage urges to smoke for self-efficacy, and SC services, for example, “Please identify the important things in your life, which may be related to personal or family health, interpersonal relationships, finances, or others. The important thing can be the driving force for quitting smoking!” The schedule of messages was adjusted to the participants’ baseline readiness to quit (within next 7 days/ 30 days/ 60 days or undecided) as according to the Transtheoretical Model (TTM).

SC advisors interacted in real-time with smokers through IM, providing behavioral support to avoid or handle high risk situations of smoking (e.g. cigarette invitation from friends), instruct the use of NRT-S and break the habitual smoking by time-contingent messages (e.g. first cigarette in the morning). Proactive IM messages such as asking about recent progress of SC were used to initiate the conversation, for example, “During this period of time, I have heard lots of good news one after another. Some people said that they had completely quit smoking, and some had reduced smoking. How about your progress? You can share it with me.” SC advisors delivered SCT- and TTM-guided advice and actively referred the smokers if they expressed the need for SC services.

Each participant in the intervention group received a unique link to access the chatbot for tracking individual’s engagement. SC advisors proactively sent a total of 6 reminders of chatbot URL through IM every two weeks during the 12-week personalized behavioral support.

The control group received the same AWARD model as the intervention group at baseline, an established standard care model for Hong Kong community smokers. They additionally received the regular SMS messages on healthy lifestyles and reminders to participate in the follow-up surveys for quitting, with a similar frequency to the regular IM sent to the intervention group.

5-ix) Describe use parameters

Describe use parameters (e.g., intended “doses” and optimal timing for use). Clarify what instructions or recommendations were given to the user, e.g., regarding timing, frequency, heaviness of use, if any, or was the intervention used ad libitum.

	1	2	3	4	5	
subitem not at all important	○	○	○	○	◉	essential

Clear selection

Does your paper address subitem 5-ix?

Copy and paste relevant sections from the manuscript (include quotes in quotation marks "like this" to indicate direct quotes from your manuscript), or elaborate on this item by providing additional information not in the ms, or briefly explain why the item is not applicable/relevant for your study

All participants received a message once a week for initiating IM conversation. The frequency increased to once daily for the week of the targeted quit date and twice weekly for the week before and after the week of the quit date. The schedule could be adjusted as requested by smokers during IM conversation.

SC advisors interacted in real-time with smokers through IM, providing behavioral support to avoid or handle high risk situations of smoking (e.g. cigarette invitation from friends), instruct the use of NRT-S and break the habitual smoking by time-contingent messages (e.g. first cigarette in the morning). Proactive IM messages such as asking about recent progress of SC were used to initiate the conversation, for example, “During this period of time, I have heard lots of good news one after another. Some people said that they had completely quit smoking, and some had reduced smoking. How about your progress? You can share it with me.”

Each participant in the intervention group received a unique link to access the chatbot for tracking individual’s engagement. SC advisors proactively sent a total of 6 reminders of chatbot URL through IM every two weeks during the 12-week personalized behavioral support.

5-x) Clarify the level of human involvement

Clarify the level of human involvement (care providers or health professionals, also technical assistance) in the e-intervention or as co-intervention (detail number and expertise of professionals involved, if any, as well as “type of assistance offered, the timing and frequency of the support, how it is initiated, and the medium by which the assistance is delivered”. It may be necessary to distinguish between the level of human involvement required for the trial, and the level of human involvement required for a routine application outside of a RCT setting (discuss under item 21 – generalizability).

	1	2	3	4	5	
subitem not at all important	○	○	○	○	◉	essential

Clear selection

Does your paper address subitem 5-x?

Copy and paste relevant sections from the manuscript (include quotes in quotation marks "like this" to indicate direct quotes from your manuscript), or elaborate on this item by providing additional information not in the ms, or briefly explain why the item is not applicable/relevant for your study

As an extension of the AWARD model at baseline, the intervention group received 12-week personalized behavioral support delivered through SC advisor-delivered IM and a fully automated chatbot.

5-xi) Report any prompts/reminders used

Report any prompts/reminders used: Clarify if there were prompts (letters, emails, phone calls, SMS) to use the application, what triggered them, frequency etc. It may be necessary to distinguish between the level of prompts/reminders required for the trial, and the level of prompts/reminders for a routine application outside of a RCT setting (discuss under item 21 – generalizability).

	1	2	3	4	5	
subitem not at all important	○	○	○	○	◉	essential

Clear selection

Does your paper address subitem 5-xi? *

Copy and paste relevant sections from the manuscript (include quotes in quotation marks "like this" to indicate direct quotes from your manuscript), or elaborate on this item by providing additional information not in the ms, or briefly explain why the item is not applicable/relevant for your study

SC advisors proactively sent a total of 6 reminders of chatbot URL through IM every two weeks during the 12-week personalized behavioral support.

5-xii) Describe any co-interventions (incl. training/support)

Describe any co-interventions (incl. training/support): Clearly state any interventions that are provided in addition to the targeted eHealth intervention, as ehealth intervention may not be designed as stand-alone intervention. This includes training sessions and support [1]. It may be necessary to distinguish between the level of training required for the trial, and the level of training for a routine application outside of a RCT setting (discuss under item 21 – generalizability.

	1	2	3	4	5	
subitem not at all important	○	○	○	○	◉	essential

Clear selection

Does your paper address subitem 5-xii? *

Copy and paste relevant sections from the manuscript (include quotes in quotation marks "like this" to indicate direct quotes from your manuscript), or elaborate on this item by providing additional information not in the ms, or briefly explain why the item is not applicable/relevant for your study

Both groups received brief advice using the AWARD model (Ask, Warning, Advice, Referral, Do-it-again) 15 at baseline. Participants were asked about smoking behaviors (Ask) and invited for an exhaled carbon monoxide test. The results were used to warn about the harms of continued smoking together with a leaflet (eFigure 1 in Supplement 2) containing shocking pictures of smoking-induced diseases (Warn). Participants were advised to quit promptly using NRT or SC services (Advise) and offered referral to a free SC service (Refer). Contacts of the participants were sent to the SC service providers of their choice for further treatment (active referral).15 The above advice was repeated during follow-ups (Do-it-again).

The intervention group additionally received 1-week free NRT-S (Nicotinell; GlaxoSmithKline, Brentford, London, UK) in the original packing (7 NRT patches or 84 pieces of gum). Our previous trial found no difference in quit rates between 1-week or 2-week NRT-S.22 The dose of the NRT-S was assigned based on the time to the first cigarette of the day.23 Participants who had their first cigarettes > 30 minutes after waking up and had not previously used NRT received 2 mg nicotine gum or 14 mg nicotine patch, while those who smoked ≤ 30 minutes were given 21 mg nicotine patch (4 mg NRT gum is not available in Hong Kong). The research assistant and trained SC advisors briefly instructed the participants on the usage of NRT and gave an instruction card (eFigure 2 in Supplement 2) containing information on NRT use and potential side effects.

6a) Completely defined pre-specified primary and secondary outcome measures, including how and when they were assessed

Does your paper address CONSORT subitem 6a? *

Copy and paste relevant sections from the manuscript (include quotes in quotation marks "like this" to indicate direct quotes from your manuscript), or elaborate on this item by providing additional information not in the ms, or briefly explain why the item is not applicable/relevant for your study

Data were collected in person at baseline and through telephone interviews at 3, 6, and 12months after randomization (intervention initiation). The primary outcomes were carbon monoxide-validated (<4 ppm) smoking abstinence at 6 and 12 months after intervention initiation.27 Participants who reported having quit tobacco use for 7 days or longer at the 6- and 12-month follow-ups were invited for breath carbon monoxide tests. Those who agreed to the tests were given HK $300 (approximately US $38) in cash for their time and traveling expense.

Secondary outcomes included self-reported 7-day point prevalence and 24-week continuous abstinences; quit attempts; smoking reduction, defined as self-reported reduction in number of cigarettes per day by at least 50% of the baseline amount; and SC service use, defined as having attended at least one treatment session delivered by a SC service provider, at 6 and 12 months. The experienced research assistant or trained SC advisors measured participants' quality of life using the five-level EuroQol five-dimensional questionnaire (EQ-5D-5L) twice at baseline and 12 months. EQ-5D-5L has been validated in Chinese, with responses transformed using the standard Hong Kong value set form ranging from -0.864, the worst to 1, the best.

6a-i) Online questionnaires: describe if they were validated for online use and apply CHERRIES items to describe how the questionnaires were designed/deployed

If outcomes were obtained through online questionnaires, describe if they were validated for online use and apply CHERRIES items to describe how the questionnaires were designed/deployed [9].

	1	2	3	4	5	
subitem not at all important	○	○	○	●	○	essential

Clear selection

Does your paper address subitem 6a-i?

Copy and paste relevant sections from manuscript text

The study did not use online questionnaires.

6a-ii) Describe whether and how “use” (including intensity of use/dosage) was defined/measured/monitored

Describe whether and how “use” (including intensity of use/dosage) was defined/measured/monitored (logins, logfile analysis, etc.). Use/adoption metrics are important process outcomes that should be reported in any ehealth trial.

	1	2	3	4	5	
subitem not at all important	○	○	○	○	●	essential

Clear selection

Does your paper address subitem 6a-ii?

Copy and paste relevant sections from manuscript text

In the intervention group, we examined the associations of intervention engagement, defined by IM/chatbot use (verified by WhatsApp conversation log9 and chatbot backend), self-reported use of NRT-S at 3 months, or both, with validated abstinence outcomes, adjusting for established predictors of SC outcomes, including sex, age, nicotine dependence, previous quit attempt, and readiness to quit.

6a-iii) Describe whether, how, and when qualitative feedback from participants was obtained

Describe whether, how, and when qualitative feedback from participants was obtained (e.g., through emails, feedback forms, interviews, focus groups).

	1	2	3	4	5	
subitem not at all important	○	○	○	●	○	essential

Clear selection

Does your paper address subitem 6a-iii?

Copy and paste relevant sections from manuscript text

We conducted post-hoc qualitative interviews in chatbot users after the complete of the trial and reported results elsewhere.

6b) Any changes to trial outcomes after the trial commenced, with reasons

Does your paper address CONSORT subitem 6b? *

Copy and paste relevant sections from the manuscript (include quotes in quotation marks "like this" to indicate direct quotes from your manuscript), or elaborate on this item by providing additional information not in the ms, or briefly explain why the item is not applicable/relevant for your study

No changes to trial outcomes after the trial commenced

7a) How sample size was determined

NPT: When applicable, details of whether and how the clustering by care provides or centers was addressed

7a-i) Describe whether and how expected attrition was taken into account when calculating the sample size

Describe whether and how expected attrition was taken into account when calculating the sample size.

	1	2	3	4	5	
subitem not at all important	○	○	○	○	◉	essential

Clear selection

Does your paper address subitem 7a-i?

Copy and paste relevant sections from manuscript title (include quotes in quotation marks "like this" to indicate direct quotes from your manuscript), or elaborate on this item by providing additional information not in the ms, or briefly explain why the item is not applicable/relevant for your study

Sample size was estimated based on our previous trial, which found that the group receiving brief advice and active referral had a 6-month biochemically validated abstinence rate of 9.0% by intention-to-treat analysis. Given an assumed effect size of 1.8 derived from a meta-analysis of mHealth SC RCTs (RR=1.83), power of 80% and an allocation ratio of 1:1, the required sample size for detecting a significant difference in biochemically validated abstinence rates between the intervention group and control group at two-sided type I error of 0.05 is 664 (each group 332).

7b) When applicable, explanation of any interim analyses and stopping guidelines

Does your paper address CONSORT subitem 7b? *

Copy and paste relevant sections from the manuscript (include quotes in quotation marks "like this" to indicate direct quotes from your manuscript), or elaborate on this item by providing additional information not in the ms, or briefly explain why the item is not applicable/relevant for your study

Not applicable to our trial.

8a) Method used to generate the random allocation sequence

NPT: When applicable, how care providers were allocated to each trial group

Does your paper address CONSORT subitem 8a? *

Copy and paste relevant sections from the manuscript (include quotes in quotation marks "like this" to indicate direct quotes from your manuscript), or elaborate on this item by providing additional information not in the ms, or briefly explain why the item is not applicable/relevant for your study

The randomization sequence with a 1:1 allocation ratio and permuted block of 4, 8, or 12 was generated by a non-investigator. Sequentially numbered, opaque, sealed envelopes (SNOSE) were prepared by an investigator not involved in participant enrolment for allocation concealment. Once a smoker signed the consent form, one SNOSE was opened according to the serial number to determine the group allocation. Masking of participants, the research assistant, and SC advisors was not possible due to the nature of behavioral interventions. Statistical analysts were blinded from the group allocation.

8b) Type of randomisation; details of any restriction (such as blocking and block size)

Does your paper address CONSORT subitem 8b? *

Copy and paste relevant sections from the manuscript (include quotes in quotation marks "like this" to indicate direct quotes from your manuscript), or elaborate on this item by providing additional information not in the ms, or briefly explain why the item is not applicable/relevant for your study

The randomization sequence with a 1:1 allocation ratio and permuted block of 4, 8, or 12 was generated by a non-investigator. Sequentially numbered, opaque, sealed envelopes (SNOSE) were prepared by an investigator not involved in participant enrolment for allocation concealment. Once a smoker signed the consent form, one SNOSE was opened according to the serial number to determine the group allocation. Masking of participants, the research assistant, and SC advisors was not possible due to the nature of behavioral interventions. Statistical analysts were blinded from the group allocation.

9) Mechanism used to implement the random allocation sequence (such as sequentially numbered containers), describing any steps taken to conceal the sequence until interventions were assigned

Does your paper address CONSORT subitem 9? *

Copy and paste relevant sections from the manuscript (include quotes in quotation marks "like this" to indicate direct quotes from your manuscript), or elaborate on this item by providing additional information not in the ms, or briefly explain why the item is not applicable/relevant for your study

The randomization sequence with a 1:1 allocation ratio and permuted block of 4, 8, or 12 was generated by a non-investigator. Sequentially numbered, opaque, sealed envelopes (SNOSE) were prepared by an investigator not involved in participant enrolment for allocation concealment. Once a smoker signed the consent form, one SNOSE was opened according to the serial number to determine the group allocation. Masking of participants, the research assistant, and SC advisors was not possible due to the nature of behavioral interventions. Statistical analysts were blinded from the group allocation.

10) Who generated the random allocation sequence, who enrolled participants, and who assigned participants to interventions

Does your paper address CONSORT subitem 10? *

Copy and paste relevant sections from the manuscript (include quotes in quotation marks "like this" to indicate direct quotes from your manuscript), or elaborate on this item by providing additional information not in the ms, or briefly explain why the item is not applicable/relevant for your study

The randomization sequence with a 1:1 allocation ratio and permuted block of 4, 8, or 12 was generated by a non-investigator.
An experienced research assistant and trained SC advisors enrolled participants and assigned participants to interventions.

11a) If done, who was blinded after assignment to interventions (for example, participants, care providers, those assessing outcomes) and how

NPT: Whether or not administering co-interventions were blinded to group assignment

11a-i) Specify who was blinded, and who wasn't

Specify who was blinded, and who wasn't. Usually, in web-based trials it is not possible to blind the participants [1, 3] (this should be clearly acknowledged), but it may be possible to blind outcome assessors, those doing data analysis or those administering co-interventions (if any).

	1	2	3	4	5	
subitem not at all important	○	○	○	○	●	essential

Clear selection

Does your paper address subitem 11a-i? *

Copy and paste relevant sections from the manuscript (include quotes in quotation marks "like this" to indicate direct quotes from your manuscript), or elaborate on this item by providing additional information not in the ms, or briefly explain why the item is not applicable/relevant for your study

Masking of participants, the research assistant, and SC advisors was not possible due to the nature of behavioral interventions. Statistical analysts were blinded from the group allocation.

11a-ii) Discuss e.g., whether participants knew which intervention was the "intervention of interest" and which one was the "comparator"

Informed consent procedures (4a-ii) can create biases and certain expectations - discuss e.g., whether participants knew which intervention was the "intervention of interest" and which one was the "comparator".

	1	2	3	4	5	
subitem not at all important	○	○	○	◉	○	essential

Clear selection

Does your paper address subitem 11a-ii?

Copy and paste relevant sections from the manuscript (include quotes in quotation marks "like this" to indicate direct quotes from your manuscript), or elaborate on this item by providing additional information not in the ms, or briefly explain why the item is not applicable/relevant for your study

Your answer

11b) If relevant, description of the similarity of interventions

(this item is usually not relevant for ehealth trials as it refers to similarity of a placebo or sham intervention to a active medication/intervention)

Does your paper address CONSORT subitem 11b? *

Copy and paste relevant sections from the manuscript (include quotes in quotation marks "like this" to indicate direct quotes from your manuscript), or elaborate on this item by providing additional information not in the ms, or briefly explain why the item is not applicable/relevant for your study

Not relevant for the present study.

12a) Statistical methods used to compare groups for primary and secondary outcomes

NPT: When applicable, details of whether and how the clustering by care providers or centers was addressed

Does your paper address CONSORT subitem 12a? *

Copy and paste relevant sections from the manuscript (include quotes in quotation marks "like this" to indicate direct quotes from your manuscript), or elaborate on this item by providing additional information not in the ms, or briefly explain why the item is not applicable/relevant for your study

All analyses were performed according to a prespecified statistical analysis plan. Primary analyses were by intention-to-treat, assuming participants with missing outcomes to have had no change in smoking behaviors from baseline. Logistic regression was used to compare the SC outcomes between groups. Planned sensitivity analyses were conducted for primary analyses. First, complete case analyses were conducted by excluding participants lost to follow-up. Second, multiple imputation by chained equations assuming data were missing at random was conducted. The imputation models included the outcomes, group allocation, and sociodemographic and baseline smoking-related characteristics that were associated with abstinence or missingness, including sex, age, highest educational attainment, monthly household income, daily cigarette consumption, time to the first cigarette after waking, previous quit attempt, and readiness to quit. Fifty imputed datasets were generated and results were pooled according to Rubin's rule.

We conducted a priori subgroup analyses by baseline characteristics, including sex, age group, education level, nicotine dependence level, any previous quit attempt, and readiness to quit in 30 days. Multiplicative interaction terms of baseline characteristics × group allocation were included in logistic regression models to calculate the P values for interaction, although the study was not powered to examine interaction. In the intervention group, we examined the associations of intervention engagement, defined by IM/chatbot use (verified by WhatsApp conversation log and chatbot backend), self-reported use of NRT-S at 3 months, or both, with validated abstinence outcomes, adjusting for established predictors of SC outcomes, including sex, age, nicotine dependence, previous quit attempt, and readiness to quit. All analyses were conducted in Stata/MP version 15.1. A 2-tailed P<0.05 indicated statistical significance.

12a-i) Imputation techniques to deal with attrition / missing values

Imputation techniques to deal with attrition / missing values: Not all participants will use the intervention/comparator as intended and attrition is typically high in ehealth trials. Specify how participants who did not use the application or dropped out from the trial were treated in the statistical analysis (a complete case analysis is strongly discouraged, and simple imputation techniques such as LOCF may also be problematic [4]).

	1	2	3	4	5	
subitem not at all important	○	○	○	○	◉	essential

Clear selection

Does your paper address subitem 12a-i? *

Copy and paste relevant sections from the manuscript (include quotes in quotation marks "like this" to indicate direct quotes from your manuscript), or elaborate on this item by providing additional information not in the ms, or briefly explain why the item is not applicable/relevant for your study

multiple imputation by chained equations assuming data were missing at random was conducted. The imputation models included the outcomes, group allocation, and sociodemographic and baseline smoking-related characteristics that were associated with abstinence or missingness, including sex, age, highest educational attainment, monthly household income, daily cigarette consumption, time to the first cigarette after waking, previous quit attempt, and readiness to quit. Fifty imputed datasets were generated and results were pooled according to Rubin's rule.

12b) Methods for additional analyses, such as subgroup analyses and adjusted analyses

Does your paper address CONSORT subitem 12b? *

Copy and paste relevant sections from the manuscript (include quotes in quotation marks "like this" to indicate direct quotes from your manuscript), or elaborate on this item by providing additional information not in the ms, or briefly explain why the item is not applicable/relevant for your study

We conducted a priori subgroup analyses by baseline characteristics, including sex, age group, education level, nicotine dependence level, any previous quit attempt, and readiness to quit in 30 days. Multiplicative interaction terms of baseline characteristics × group allocation were included in logistic regression models to calculate the P values for interaction, although the study was not powered to examine interaction. In the intervention group, we examined the associations of intervention engagement, defined by IM/chatbot use (verified by WhatsApp conversation log and chatbot backend), self-reported use of NRT-S at 3 months, or both, with validated abstinence outcomes, adjusting for established predictors of SC outcomes, including sex, age, nicotine dependence, previous quit attempt, and readiness to quit. All analyses were conducted in Stata/MP version 15.1. A 2-tailed P<0.05 indicated statistical significance.

X26) REB/IRB Approval and Ethical Considerations [recommended as subheading under "Methods"] (not a CONSORT item)

X26-i) Comment on ethics committee approval

	1	2	3	4	5	
subitem not at all important	○	○	○	○	◉	essential

Clear selection

Does your paper address subitem X26-i?

Copy and paste relevant sections from the manuscript (include quotes in quotation marks "like this" to indicate direct quotes from your manuscript), or elaborate on this item by providing additional information not in the ms, or briefly explain why the item is not applicable/relevant for your study

Ethical approval was granted by the Institutional Review Board of the University of Hong Kong/ Hospital Authority Hong Kong West Cluster (UW 18–405).

x26-ii) Outline informed consent procedures

Outline informed consent procedures e.g., if consent was obtained offline or online (how? Checkbox, etc.?), and what information was provided (see 4a-ii). See [6] for some items to be included in informed consent documents.

	1	2	3	4	5	
subitem not at all important	○	○	○	○	◉	essential

Clear selection

Does your paper address subitem X26-ii?

Copy and paste relevant sections from the manuscript (include quotes in quotation marks "like this" to indicate direct quotes from your manuscript), or elaborate on this item by providing additional information not in the ms, or briefly explain why the item is not applicable/relevant for your study

All participants provided written informed consent.

X26-iii) Safety and security procedures

Safety and security procedures, incl. privacy considerations, and any steps taken to reduce the likelihood or detection of harm (e.g., education and training, availability of a hotline)

	1	2	3	4	5	
subitem not at all important	○	○	○	○	◉	essential

Clear selection

Does your paper address subitem X26-iii?

Copy and paste relevant sections from the manuscript (include quotes in quotation marks "like this" to indicate direct quotes from your manuscript), or elaborate on this item by providing additional information not in the ms, or briefly explain why the item is not applicable/relevant for your study

Instructions and support were provided to participants, and no adverse symptoms were reported at follow-up.

RESULTS

13a) For each group, the numbers of participants who were randomly assigned, received intended treatment, and were analysed for the primary outcome

NPT: The number of care providers or centers performing the intervention in each group and the number of patients treated by each care provider in each center

Does your paper address CONSORT subitem 13a? *

Copy and paste relevant sections from the manuscript (include quotes in quotation marks "like this" to indicate direct quotes from your manuscript), or elaborate on this item by providing additional information not in the ms, or briefly explain why the item is not applicable/relevant for your study

Figure 1 shows that, of 711 smokers screened for eligibility, 664 participants were individually randomized. The retention rate was 69.9%, 67.2%, 73.2% at 3, 6, and 12 months, respectively. Retention rates were similar between the 2 groups (P=0.49–0.95).

13b) For each group, losses and exclusions after randomisation, together with reasons

Does your paper address CONSORT subitem 13b? (NOTE: Preferably, this is shown in a CONSORT flow diagram) *

Copy and paste relevant sections from the manuscript (include quotes in quotation marks "like this" to indicate direct quotes from your manuscript), or elaborate on this item by providing additional information not in the ms, or briefly explain why the item is not applicable/relevant for your study

Figure 1 shows that, of 711 smokers screened for eligibility, 664 participants were individually randomized. The retention rate was 69.9%, 67.2%, 73.2% at 3, 6, and 12 months, respectively. Retention rates were similar between the 2 groups (P=0.49–0.95).

13b-i) Attrition diagram

Strongly recommended: An attrition diagram (e.g., proportion of participants still logging in or using the intervention/comparator in each group plotted over time, similar to a survival curve) or other figures or tables demonstrating usage/dose/engagement.

	1	2	3	4	5	
subitem not at all important	○	○	○	○	◉	essential

Clear selection

Does your paper address subitem 13b-i?

Copy and paste relevant sections from the manuscript or cite the figure number if applicable (include quotes in quotation marks "like this" to indicate direct quotes from your manuscript), or elaborate on this item by providing additional information not in the ms, or briefly explain why the item is not applicable/relevant for your study

Intervention engagement
In the intervention group, 33.1% (110/332) had used mHealth technologies (IM only: 22.3%, 74/332; chatbot only: 4.0%, 13/332; both IM and chatbot: 7.0%, 23/332) and 25.6% (85/332) had used NRT-S by 3 months. Table 4 shows that, compared with no engagement in IM or Chatbot, engagement in IM only showed significantly higher ORs of validated abstinence at 6 months (adjusted OR [AOR]=4.71, 95% CI 1.24, 17.81) after adjusting for baseline characteristics, and the OR further increased for engagement in both IM and Chatbot (AOR=8.95, 95% CI 1.79, 44.75). Of 85 participants who used NRT-S, 67.1% reported no side effect, while 11.8% reported headache/dizziness and 8.3% reported skin problems (eTable 2 in Supplement 2). Instructions and support were provided to participants, and no adverse symptoms were reported at follow-up.

14a) Dates defining the periods of recruitment and follow-up

Does your paper address CONSORT subitem 14a? *

Copy and paste relevant sections from the manuscript (include quotes in quotation marks "like this" to indicate direct quotes from your manuscript), or elaborate on this item by providing additional information not in the ms, or briefly explain why the item is not applicable/relevant for your study

Your answer

14a-i) Indicate if critical "secular events" fell into the study period

Indicate if critical "secular events" fell into the study period, e.g., significant changes in Internet resources available or "changes in computer hardware or Internet delivery resources"

	1	2	3	4	5	
subitem not at all important	○	○	◉	○	○	essential

Clear selection

Does your paper address subitem 14a-i?

Copy and paste relevant sections from the manuscript (include quotes in quotation marks "like this" to indicate direct quotes from your manuscript), or elaborate on this item by providing additional information not in the ms, or briefly explain why the item is not applicable/relevant for your study

No critical "secular events" fell into the study period.

14b) Why the trial ended or was stopped (early)

Does your paper address CONSORT subitem 14b? *

Copy and paste relevant sections from the manuscript (include quotes in quotation marks "like this" to indicate direct quotes from your manuscript), or elaborate on this item by providing additional information not in the ms, or briefly explain why the item is not applicable/relevant for your study

The trial did not end or stop early.

15) A table showing baseline demographic and clinical characteristics for each group

NPT: When applicable, a description of care providers (case volume, qualification, expertise, etc.) and centers (volume) in each group

Does your paper address CONSORT subitem 15? *

Copy and paste relevant sections from the manuscript (include quotes in quotation marks "like this" to indicate direct quotes from your manuscript), or elaborate on this item by providing additional information not in the ms, or briefly explain why the item is not applicable/relevant for your study

Please see Table 1 on baseline demographic and clinical characteristics for each group.

15-i) Report demographics associated with digital divide issues

In ehealth trials it is particularly important to report demographics associated with digital divide issues, such as age, education, gender, social-economic status, computer/Internet/ehealth literacy of the participants, if known.

	1	2	3	4	5	
subitem not at all important	○	○	◉	○	○	essential

Clear selection

Does your paper address subitem 15-i? *

Copy and paste relevant sections from the manuscript (include quotes in quotation marks "like this" to indicate direct quotes from your manuscript), or elaborate on this item by providing additional information not in the ms, or briefly explain why the item is not applicable/relevant for your study

Not reported and has been acknowledged in the limitations.

16) For each group, number of participants (denominator) included in each analysis and whether the analysis was by original assigned groups

16-i) Report multiple "denominators" and provide definitions

Report multiple "denominators" and provide definitions: Report N's (and effect sizes) "across a range of study participation [and use] thresholds" [1], e.g., N exposed, N consented, N used more than x times, N used more than y weeks, N participants "used" the intervention/comparator at specific pre-defined time points of interest (in absolute and relative numbers per group). Always clearly define "use" of the intervention.

	1	2	3	4	5	
subitem not at all important	○	○	○	○	◉	essential

Clear selection

Does your paper address subitem 16-i? *

Copy and paste relevant sections from the manuscript (include quotes in quotation marks "like this" to indicate direct quotes from your manuscript), or elaborate on this item by providing additional information not in the ms, or briefly explain why the item is not applicable/relevant for your study

In the intervention group, 33.1% (110/332) had used mHealth technologies (IM only: 22.3%, 74/332; chatbot only: 4.0%, 13/332; both IM and chatbot: 7.0%, 23/332) and 25.6% (85/332) had used NRT-S by 3 months. Table 4 shows that, compared with no engagement in IM or Chatbot, engagement in IM only showed significantly higher ORs of validated abstinence at 6 months (adjusted OR [AOR]=4.71, 95% CI 1.24, 17.81) after adjusting for baseline characteristics, and the OR further increased for engagement in both IM and Chatbot (AOR=8.95, 95% CI 1.79, 44.75).

16-ii) Primary analysis should be intent-to-treat

Primary analysis should be intent-to-treat, secondary analyses could include comparing only “users”, with the appropriate caveats that this is no longer a randomized sample (see 18-i).

	1	2	3	4	5	
subitem not at all important	○	○	○	○	◉	essential

Clear selection

Does your paper address subitem 16-ii?

Copy and paste relevant sections from the manuscript (include quotes in quotation marks "like this" to indicate direct quotes from your manuscript), or elaborate on this item by providing additional information not in the ms, or briefly explain why the item is not applicable/relevant for your study

Table 2 shows that, by intention-to-treat, the intervention group did not significantly increase biochemically validated abstinence at 6 months (3.9% vs 3.0%; OR=1.31, 95% CI 0.57, 3.04) and 12 months (5.4% vs 4.5%; OR=1.21, 95% CI 0.60, 2.45). Non-significant increases were also shown in self-reported 7-day point-prevalence abstinence, smoking reduction, and use of SC service at 6 and 12 months. The intervention group showed significantly higher rates of quit attempts at 6 months than the control group (47.0% vs 38.0%; OR=1.45, 95% CI 1.06, 1.97). Sensitivity analyses using multiple imputation and complete case analyses yielded similar results (eTable 1 in Supplement 2).

17a) For each primary and secondary outcome, results for each group, and the estimated effect size and its precision (such as 95% confidence interval)

Does your paper address CONSORT subitem 17a? *

Copy and paste relevant sections from the manuscript (include quotes in quotation marks "like this" to indicate direct quotes from your manuscript), or elaborate on this item by providing additional information not in the ms, or briefly explain why the item is not applicable/relevant for your study

Table 2 shows that, by intention-to-treat, the intervention group did not significantly increase biochemically validated abstinence at 6 months (3.9% vs 3.0%; OR=1.31, 95% CI 0.57, 3.04) and 12 months (5.4% vs 4.5%; OR=1.21, 95% CI 0.60, 2.45). Non-significant increases were also shown in self-reported 7-day point-prevalence abstinence, smoking reduction, and use of SC service at 6 and 12 months. The intervention group showed significantly higher rates of quit attempts at 6 months than the control group (47.0% vs 38.0%; OR=1.45, 95% CI 1.06, 1.97). Sensitivity analyses using multiple imputation and complete case analyses yielded similar results (eTable 1 in Supplement 2).

17a-i) Presentation of process outcomes such as metrics of use and intensity of use

In addition to primary/secondary (clinical) outcomes, the presentation of process outcomes such as metrics of use and intensity of use (dose, exposure) and their operational definitions is critical. This does not only refer to metrics of attrition (13-b) (often a binary variable), but also to more continuous exposure metrics such as “average session length”. These must be accompanied by a technical description how a metric like a “session” is defined (e.g., timeout after idle time) [1] (report under item 6a).

	1	2	3	4	5	
subitem not at all important	○	○	○	○	◉	essential

Clear selection

Does your paper address subitem 17a-i?

Copy and paste relevant sections from the manuscript (include quotes in quotation marks "like this" to indicate direct quotes from your manuscript), or elaborate on this item by providing additional information not in the ms, or briefly explain why the item is not applicable/relevant for your study

In the intervention group, 33.1% (110/332) had used mHealth technologies (IM only: 22.3%, 74/332; chatbot only: 4.0%, 13/332; both IM and chatbot: 7.0%, 23/332) and 25.6% (85/332) had used NRT-S by 3 months. Table 4 shows that, compared with no engagement in IM or Chatbot, engagement in IM only showed significantly higher ORs of validated abstinence at 6 months (adjusted OR [AOR]=4.71, 95% CI 1.24, 17.81) after adjusting for baseline characteristics, and the OR further increased for engagement in both IM and Chatbot (AOR=8.95, 95% CI 1.79, 44.75).

17b) For binary outcomes, presentation of both absolute and relative effect sizes is recommended

Does your paper address CONSORT subitem 17b? *

Copy and paste relevant sections from the manuscript (include quotes in quotation marks "like this" to indicate direct quotes from your manuscript), or elaborate on this item by providing additional information not in the ms, or briefly explain why the item is not applicable/relevant for your study

Table 2 shows that, by intention-to-treat, the intervention group did not significantly increase biochemically validated abstinence at 6 months (3.9% vs 3.0%; OR=1.31, 95% CI 0.57, 3.04) and 12 months (5.4% vs 4.5%; OR=1.21, 95% CI 0.60, 2.45). Non-significant increases were also shown in self-reported 7-day point-prevalence abstinence, smoking reduction, and use of SC service at 6 and 12 months. The intervention group showed significantly higher rates of quit attempts at 6 months than the control group (47.0% vs 38.0%; OR=1.45, 95% CI 1.06, 1.97). Sensitivity analyses using multiple imputation and complete case analyses yielded similar results (eTable 1 in Supplement 2).

18) Results of any other analyses performed, including subgroup analyses and adjusted analyses, distinguishing pre-specified from exploratory

Does your paper address CONSORT subitem 18? *

Copy and paste relevant sections from the manuscript (include quotes in quotation marks "like this" to indicate direct quotes from your manuscript), or elaborate on this item by providing additional information not in the ms, or briefly explain why the item is not applicable/relevant for your study

Subgroup analyses
Table 3 shows that the intervention effect was greater in females (8.4% vs 2.3%; OR=3.91, 95% CI 0.79, 19.42) than in males (4.4% vs 5.3%, OR=0.82, 95% CI 0.36, 1.88) at 12 months and in those who were not ready to quit in 30 days (3.9% vs 1.1%; OR=3.70, 95% CI 0.74, 18.60) than those who were ready to quit in 30 days (4.0% vs 5.5%; OR=0.71, 95% CI 0.25, 2.01) at 6 months with marginal significance of interaction (both P=0.09). Although all interaction effects were not significant (probably due to small sample size), those who were female, aged 18–29 years, with lower education level (secondary or below), light nicotine dependence, no previous quit attempt, and not ready to quit in 30 days showed greater ORs of quitting at 6 and 12 months.

Intervention engagement
In the intervention group, 33.1% (110/332) had used mHealth technologies (IM only: 22.3%, 74/332; chatbot only: 4.0%, 13/332; both IM and chatbot: 7.0%, 23/332) and 25.6% (85/332) had used NRT-S by 3 months. Table 4 shows that, compared with no engagement in IM or Chatbot, engagement in IM only showed significantly higher ORs of validated abstinence at 6 months (adjusted OR [AOR]=4.71, 95% CI 1.24, 17.81) after adjusting for baseline characteristics, and the OR further increased for engagement in both IM and Chatbot (AOR=8.95, 95% CI 1.79, 44.75). Of 85 participants who used NRT-S, 67.1% reported no side effect, while 11.8% reported headache/dizziness and 8.3% reported skin problems (eTable 2 in Supplement 2). Instructions and support were provided to participants, and no adverse symptoms were reported at follow-up.

18-i) Subgroup analysis of comparing only users

A subgroup analysis of comparing only users is not uncommon in ehealth trials, but if done, it must be stressed that this is a self-selected sample and no longer an unbiased sample from a randomized trial (see 16-iii).

	1	2	3	4	5	
subitem not at all important	○	○	○	○	◉	essential

Clear selection

Does your paper address subitem 18-i?

Copy and paste relevant sections from the manuscript (include quotes in quotation marks "like this" to indicate direct quotes from your manuscript), or elaborate on this item by providing additional information not in the ms, or briefly explain why the item is not applicable/relevant for your study

We did not conduct subgroup analysis of comparing only users.

19) All important harms or unintended effects in each group

(for specific guidance see CONSORT for harms)

Does your paper address CONSORT subitem 19? *

Copy and paste relevant sections from the manuscript (include quotes in quotation marks "like this" to indicate direct quotes from your manuscript), or elaborate on this item by providing additional information not in the ms, or briefly explain why the item is not applicable/relevant for your study

Of 85 participants who used NRT-S, 67.1% reported no side effect, while 11.8% reported headache/dizziness and 8.3% reported skin problems (eTable 2 in Supplement 2). Instructions and support were provided to participants, and no adverse symptoms were reported at follow-up.

19-i) Include privacy breaches, technical problems

Include privacy breaches, technical problems. This does not only include physical "harm" to participants, but also incidents such as perceived or real privacy breaches [1], technical problems, and other unexpected/unintended incidents. "Unintended effects" also includes unintended positive effects [2].

	1	2	3	4	5	
subitem not at all important	○	○	○	○	○	essential

Does your paper address subitem 19-i?

Copy and paste relevant sections from the manuscript (include quotes in quotation marks "like this" to indicate direct quotes from your manuscript), or elaborate on this item by providing additional information not in the ms, or briefly explain why the item is not applicable/relevant for your study

None was reported in our trial.

19-ii) Include qualitative feedback from participants or observations from staff/researchers

Include qualitative feedback from participants or observations from staff/researchers, if available, on strengths and shortcomings of the application, especially if they point to unintended/unexpected effects or uses. This includes (if available) reasons for why people did or did not use the application as intended by the developers.

	1	2	3	4	5	
subitem not at all important	○	○	○	◉	○	essential

Clear selection

Does your paper address subitem 19-ii?

Copy and paste relevant sections from the manuscript (include quotes in quotation marks "like this" to indicate direct quotes from your manuscript), or elaborate on this item by providing additional information not in the ms, or briefly explain why the item is not applicable/relevant for your study

We conducted post-hoc qualitative interviews in chatbot users after the complete of the trial and reported results elsewhere.
References: Guo Z, Lee JJ, Guo N, et al. Community smokers' experiences of chatbot and chat-based instant messaging support for smoking cessation. Int J Qual Methods.2021; 25 (20), 22-23.

DISCUSSION

22) Interpretation consistent with results, balancing benefits and harms, and considering other relevant evidence
NPT: In addition, take into account the choice of the comparator, lack of or partial blinding, and unequal expertise of care providers or centers in each group

22-i) Restate study questions and summarize the answers suggested by the data, starting with primary outcomes and process outcomes (use)
Restate study questions and summarize the answers suggested by the data, starting with primary outcomes and process outcomes (use).

	1	2	3	4	5	
subitem not at all important	○	○	○	○	◉	essential

Clear selection

Does your paper address subitem 22-i? *
Copy and paste relevant sections from the manuscript (include quotes in quotation marks "like this" to indicate direct quotes from your manuscript), or elaborate on this item by providing additional information not in the ms, or briefly explain why the item is not applicable/relevant for your study

This pragmatic RCT found that behavioral support delivered through IM and chatbot combined with NRT-S compared with SMS on general health did not significantly improve validated abstinence (primary outcome), self-reported 7-day point-prevalence abstinence, smoking reduction, and use of SC services at 6 and 12 months, in proactively recruited community smokers in Hong Kong. However, engagement with the combined intervention of behavioral support through IM, chatbot, and NRT-S was low in the intervention group (11.4%, 38/332).

22-ii) Highlight unanswered new questions, suggest future research
Highlight unanswered new questions, suggest future research.

	1	2	3	4	5	
subitem not at all important	○	○	○	○	◉	essential

Clear selection

Does your paper address subitem 22-ii?
Copy and paste relevant sections from the manuscript (include quotes in quotation marks "like this" to indicate direct quotes from your manuscript), or elaborate on this item by providing additional information not in the ms, or briefly explain why the item is not applicable/relevant for your study

Engagement has been a major challenge for mHealth interventions particularly for those not ready for behavior change. Future trials on mHealth SC support may balance the busy schedule of participants by extending IM-based service hours. Future SC chatbots could incorporate artificial intelligence techniques such as natural language processing and machine learning to better simulate human-to-human interaction.

20) Trial limitations, addressing sources of potential bias, imprecision, and, if relevant, multiplicity of analyses

20-i) Typical limitations in ehealth trials

Typical limitations in ehealth trials: Participants in ehealth trials are rarely blinded. Ehealth trials often look at a multiplicity of outcomes, increasing risk for a Type I error. Discuss biases due to non-use of the intervention/usability issues, biases through informed consent procedures, unexpected events.

	1	2	3	4	5	
subitem not at all important	○	○	○	○	◉	essential

Clear selection

Does your paper address subitem 20-i? *

Copy and paste relevant sections from the manuscript (include quotes in quotation marks "like this" to indicate direct quotes from your manuscript), or elaborate on this item by providing additional information not in the ms, or briefly explain why the item is not applicable/relevant for your study

Engagement has been a major challenge for mHealth interventions particularly for those not ready for behavior change. Similarly, we found low intervention engagement (IM only: 22.3%, 74/332; chatbot only: 4.0%, 13/332; both IM and chatbot: 7.0%, 23/332).

21) Generalisability (external validity, applicability) of the trial findings

NPT: External validity of the trial findings according to the intervention, comparators, patients, and care providers or centers involved in the trial

21-i) Generalizability to other populations

Generalizability to other populations: In particular, discuss generalizability to a general Internet population, outside of a RCT setting, and general patient population, including applicability of the study results for other organizations

	1	2	3	4	5	
subitem not at all important	○	○	○	○	◉	essential

Clear selection

Does your paper address subitem 21-i?

Copy and paste relevant sections from the manuscript (include quotes in quotation marks "like this" to indicate direct quotes from your manuscript), or elaborate on this item by providing additional information not in the ms, or briefly explain why the item is not applicable/relevant for your study

Fifth, participants were mainly male and were mostly with a low to moderate level of nicotine dependence, without past quit attempts, and not ready to quit in the short term. The generalizability of our results was uncertain to other populations with different sociodemographic and smoking characteristics. Sixth, this trial may be less applicable to other interventions that do not refer to SC services or in other settings with limited SC services.

21-ii) Discuss if there were elements in the RCT that would be different in a routine application setting

Discuss if there were elements in the RCT that would be different in a routine application setting (e.g., prompts/reminders, more human involvement, training sessions or other co-interventions) and what impact the omission of these elements could have on use, adoption, or outcomes if the intervention is applied outside of a RCT setting.

	1	2	3	4	5	
subitem not at all important	○	○	○	○	◉	essential

Clear selection

Does your paper address subitem 21-ii?

Copy and paste relevant sections from the manuscript (include quotes in quotation marks "like this" to indicate direct quotes from your manuscript), or elaborate on this item by providing additional information not in the ms, or briefly explain why the item is not applicable/relevant for your study

A total of 6 reminders were proactively sent by SC advisors.

OTHER INFORMATION

23) Registration number and name of trial registry

Does your paper address CONSORT subitem 23? *

Copy and paste relevant sections from the manuscript (include quotes in quotation marks "like this" to indicate direct quotes from your manuscript), or elaborate on this item by providing additional information not in the ms, or briefly explain why the item is not applicable/relevant for your study

ClinicalTrials.gov Identifier: NCT04001972.

24) Where the full trial protocol can be accessed, if available

Does your paper address CONSORT subitem 24? *

Cite a Multimedia Appendix, other reference, or copy and paste relevant sections from the manuscript (include quotes in quotation marks "like this" to indicate direct quotes from your manuscript), or elaborate on this item by providing additional information not in the ms, or briefly explain why the item is not applicable/relevant for your study

The full trial protocol was submitted as Supplementary 1.

25) Sources of funding and other support (such as supply of drugs), role of funders

Does your paper address CONSORT subitem 25? *

Copy and paste relevant sections from the manuscript (include quotes in quotation marks "like this" to indicate direct quotes from your manuscript), or elaborate on this item by providing additional information not in the ms, or briefly explain why the item is not applicable/relevant for your study

Funding/Support: This research was funded by Health and Medical Research Fund Research Fellowship Scheme, Food and Health Bureau, Government of the Hong Kong SAR (ref no. 03170087).

Role of the Funder/Sponsor: The funder had no role in the design and conduct of the study; collection, management, analysis, and interpretation of the data; preparation, review, or approval of the manuscript; and decision to submit the manuscript for publication.

X27) Conflicts of Interest (not a CONSORT item)

X27-i) State the relation of the study team towards the system being evaluated

In addition to the usual declaration of interests (financial or otherwise), also state the relation of the study team towards the system being evaluated, i.e., state if the authors/evaluators are distinct from or identical with the developers/sponsors of the intervention.

	1	2	3	4	5	
subitem not at all important	○	○	○	○	●	essential

Clear selection

Does your paper address subitem X27-i?

Copy and paste relevant sections from the manuscript (include quotes in quotation marks "like this" to indicate direct quotes from your manuscript), or elaborate on this item by providing additional information not in the ms, or briefly explain why the item is not applicable/relevant for your study

Conflict of Interest Disclosures: None reported.

About the CONSORT EHEALTH checklist

As a result of using this checklist, did you make changes in your manuscript? *

- ● yes, major changes
- ○ yes, minor changes
- ○ no

What were the most important changes you made as a result of using this checklist?

The description of the chatbot intervention.

How much time did you spend on going through the checklist INCLUDING making changes in your manuscript *

Two days were spent on going through the checklist.

As a result of using this checklist, do you think your manuscript has improved? *

- (x) yes
- ( ) no
- ( ) Other:

Would you like to become involved in the CONSORT EHEALTH group?

This would involve for example becoming involved in participating in a workshop and writing an "Explanation and Elaboration" document

- (x) yes
- ( ) no
- ( ) Other:

Clear selection

Any other comments or questions on CONSORT EHEALTH

Thank you. We have now other comments.

STOP - Save this form as PDF before you click submit

To generate a record that you filled in this form, we recommend to generate a PDF of this page (on a Mac, simply select "print" and then select "print as PDF") before you submit it.

When you submit your (revised) paper to JMIR, please upload the PDF as supplementary file.

Don't worry if some text in the textboxes is cut off, as we still have the complete information in our database. Thank you!

Final step: Click submit !

Click submit so we have your answers in our database!

Submit

Clear form

Never submit passwords through Google Forms.

This form was created outside of your domain. Report Abuse - Terms of Service - Privacy Policy

Google Forms

A. Title:
Intervention combining interactive communication technologies and nicotine replacement therapy sampling for proactively recruited smokers in smoking hotspots: a pragmatic randomized controlled trial

B. Introduction
<u>The need for innovative and scalable intervention for promoting smoking cessation in Hong Kong</u>
Smoking is the leading cause of death worldwide and in Hong Kong (HK) and smoking cessation (SC) is among the most cost-effective medical interventions. HK has a relative low smoking prevalence (10.5% daily smokers) but further decrease is needed (to 5%) to implement tobacco endgame policies. This is very challenging as many (95%) do not actively seek for SC services, which are free in HK [1]. Proactive recruitment of smokers in community is needed as they constitute the majority of smokers. We have developed a systematic method to recruit smokers at smoking hotspots (SH, defined as public outdoor places where smokers stop/linger and smoke) and have consecutively recruited over 6000 community smokers in the past 6 years [2]. Briefly-trained healthcare (nursing, medical) students adopt "a-foot-into-the-door" strategy to approach and recruit smokers in SH: they first ask the smokers some simple questions related to their smoking and those who answered were further invited to participate in the SC trials. Our data has shown that smokers proactively recruited at SH had comparable demographic characteristics and smoking behaviours to smokers in the general population [3].

<u>Brief smoking cessation advice model</u>
Behavioral or psychological counseling is effective for SC but mostly are too and expensive to apply in clinical or community settings. Brief intervention such as 5As (Ask; Advise to quit; Assess willingness to make quit attempts; Assist in quit attempts, Arrange follow-up) is recommended for clinical practice in many countries. We have developed and validated a brief (<5 minute) SC advice intervention, the AWARD model (Ask, Warn, Advise, Refer, Do-it-again), delivered by trained health care students to smokers in the community [2, 4-9]. We propose to enhance our AWARD model (published in *JAMA Internal Medicine* [9]) by adding NRT sampling and innovative technologies including automated response system and instant messaging (IM). The novel method will be even more cost-effective and sustainable for practice.

<u>NRT sampling (NRT-S)</u>
Although NRT is effective in reducing nicotine withdrawal symptoms and increasing quit rate by 50-70%, most smokers (80% in HK) have never used NRT probably due to perceived high-cost, misperceptions and low self-efficacy. Duration of NRT treatment varied from 8 to 24 weeks and only half smokers completed the treatment. Our study showed only 16% SC clinic users used NRT for 4-week or longer [10]. As nicotine withdrawal symptoms peak at 1-week and gradually decrease 2-3 weeks after smoking abstinence, NRT use in the initial period of abstinence to combat withdrawal symptoms is most important [11]. Studies have found NRT (1-6 weeks) sampled to Quitline users increased quit attempts, quit rate, and NRT or SC service use [12]. Our RCT has found similar quit rates at 6-month follow-up in SC clinic users provided with 1 week (27.5%) or 2-week (27.3%) NRT samples [13], suggesting minimal NRT for 1-week may attract and motivate some smokers to reduce or quit smoking, use NRT for longer, and visit SC services which provide more NRT. NRT-S has now also been used to attract smokers in community to use SC services in HK (e.g. TWGHs ISCC).

<u>Technology-enhanced intervention</u>
Systematic reviews have shown text messaging-based (SMS) intervention were effective to promote abstinence among smokers with modest effect sizes ranging from 1.38-1.83 [14, 15]. Given the SMS design, smokers can only have very limited interaction with the computers [15]. More intensive, personalized and synchronous interaction can provide stronger psychosocial support to promote quitting among the smokers. Recent technological advances allows using IM apps (e.g. WhatsApp interaction with a trained counsellors or advisors) and automatic dialogue system (Conversation agent or Chatbot), which have been applied to promoting medication adherence and delivery of mental health interventions [16, 17]. Rapid development of natural language processing (NLP), machine learning through Bigdata analysis allows using Chatbot to deliver

interactive dialogues to promote health outcomes (e.g. mental health promotion [18]). Chatbot, which can be assessed 24hrs/7days, can effectively reduce manpower of IM Apps advisors for providing psychosocial support. Open source NLP tools such as NLTK (http://www.nltk.org) and machine intelligence tools such as Tensorflow (https://www.tensorflow.org) facilitate design of Chatbot for various purposes with user-friendly operating procedures. There are some existing Chatbots in social media (e.g. https://chatbottle.co/bots/stopsmoking-1 & https://chatfuel.com/bot/becomingsmokefree) for general SC advice in western context but none were designed to focus on a particular research purpose. We find no similar RCTs in the PubMed, Cochrane Library or clinical trial registries until Dec 2017. Therefore, we propose to assess the effectiveness of a technology-enhanced brief advice SC model plus NRT-S on smoking abstinence in proactively recruited smokers in HK.

Pilot studies

The proposal is supported by 3 pilot studies. **(**1) We have pilot-tested NRT-S in a RCT (ClinicalTrials.gov ID: NCT02935231) among 32 smokers recruited in SH in 2016 (8 smokers in each arm). At 1-month follow-up, more reduction in daily cigarette consumption was found in NRT-S groups (NRT-S + Advice: 4.4 ± 4.7; NRT-S only: 3.5 ± 2.4) and advice only group (5.8 ± 3.8) compared with the control group (2.3 ± 2.3). (2**)** Five focus group interviews on 21 smokers in 2017 (68% male, 55% daily smokers, mean age 49) explored their acceptability and expected intensity and frequency of IM for SC (HKHAW/HKU IRB no. UW 17-206; manuscript being prepared). IM Apps was regarded as a suitable, useful and personalized intervention to encourage smokers to quit smoking. The frequencies and duration of regular messages needed to be tailored to smokers' needs (e.g. quit date). (3**)** A trial conducted under the context of "Quit-to-Win" testing the feasibility of using WhatsApp on supporting SC among community smokers (ClinicalTrials.gov ID: NCT03182790) showed that about 95% used IM Apps daily. At 1-month follow up, among 156 smokers randomized to receive IM (AWARD baseline plus IM psychosocial support), 96% received and read our IM messages (only 3 did not read because they were not interested or too busy) and 31% chatted with the counsellors at least weekly. Many reported the messages were helpful to increase their motivation to quit (66%) and quit attempt (57%). They generally were satisfied with the interaction with the counsellor (score 8.3/10). Preliminary results showed higher self-reported quit rate in intervention (14.2%) compared with control (8.2%) (AWARD advice only) at 3-month follow-up. The unstructured text data from this trial will be used to train the proposed Chatbot.

C. Aims and Hypotheses to be Tested

(1) To assess the main effect of the Intervention vs. Control group on biochemical validated smoking abstience at 6-month and 12-month. (Primary)
(2) To assess the above main effects on secondary outcomes (see below) at 6-month and 12-month.
(3) To identify the potential mediators between intervention and outcomes.
(4) To evaluate the cost-effectiveness of the interventions.
(5) To understand the subjects' experience of IM/Chatbot support on SC.

D. Plan of Investigation

The CONSORT flow chart in appendix 1 summarizes the timeline of the proposed study.
(1) Subjects:
Inclusion criteria
- Adult smokers aged 18+ years who smoke cigarette(s) daily.
- Exhaled carbon monoxide (CO) level of ≥4ppm.
- Having smartphones and willing to install IM Apps and a Chatbot.
- Hong Kong residents able to read and communicate in Chinese (Cantonese or Putonghua).

Exclusion criteria
- Smokers who have psychiatric/psychological diseases/on regular psychotropic medications.
- Smokers who are using SC medication, NRT, other SC services or projects.
- Smokers who have contraindication for NRT use: severe angina, arrhythmia, myocardia infraction, pregnancy (or intended to become pregnant <6 months) or breastfeeding.

Settings
We aim to include a reasonably "representative" or unbiased sample of SH at different locations from all 3 major regions (HK Island, Kowloon and New Territories) of HK. A sampling frame of all SH is not available and cannot be established within this proposal. We have identified 15 hotspots and recruited 750+ smokers in 4 months in 2015. The locations of SH include exits of underground transit and railway stations, shopping malls and large commercial buildings [5]. We will select 15 more SHs from the field observation results of public open places across the 3 major regions, making up a total of 30 SH for recruitment. A trained observer will count pedestrian flow, number of smokers and assess the suitability of the environment for delivering intervention (including smokers' duration of stay, noise level, space) using improved standardized forms.

Subject recruitment
Two trained SC advisors (student helpers) and 1 supervisor (experienced research nurse) will conduct intervention in each session. Potential subjects at the SH will be approached using the "a-foot-into-the-door" strategy (see "Introduction"). If there are more than 1 potential subjects, the SC advisors will randomly select 1 smoker to avoid contamination (as they may share information from IM Apps subsequently). Smokers will be assessed for eligibility and informed written consent will be sought. Subjects will complete a brief self-administered baseline questionnaire to provide data on socio-demographic characteristics and smoking (see "Measurements"). To increase Chatbot/IM App intervention compliance and reduce later hang-ups of telephone surveys, a designated study telephone number will be saved into subjects' mobile phones. This increased response rate to 80% at 1-month in our trial in 2017.

Sample size calculation
As there is no similar trial in the literature, sample size was calculated based on our previous trials. The validated quit rate for the subjects who received AWARD advice and active referral was about 9% at 6-month & 12-month follow-up and conservative assumption of an effect size of 1.8 (usual effect size for NRT-S trials), type I error 0.05, power 80% and allocation ratio 1:1, the required sample size for determining a significant group difference of biochemically validated quit rates between Intervention group and Control group is 664 (each group 332).

(2) Methods
Intervention group
Brief advice (AWARD model)
Subjects in the intervention group will receive brief SC advice using ***AWARD*** model (***Ask***, ***W***arning, ***A***dvice, ***R***eferral and ***D****o-it-again*) to be delivered by the SC advisors using about 2 to 5 minutes. Details of ***AWARD*** are as below.
1. ***Ask***: smoking habit, quit intention and attempt, smoking reduction intention, previous quitting experience including NRT and SC use.
2. ***Warn***: smokers will be orally warned about the harms of smoking and receive a A5-sized leaflet, which includes some shocking pictures of smoking-related diseases, and SC services information (Appendix 2).
3. ***Advise***: smokers will be advised to quit as soon as possible and use NRT or SC services.
4. ***Refer***: smokers will be encouraged to seek SC services for free NRT or other SC services. Those who agreed will be actively referred to their preferred SC services. The collected contact information with consent will be sent to the SC services providers for a quick appointment and follow-up. We will liaise with all SC providers for progress monitoring and data collection on service use [9].
5. ***Do-it-again***: Relapsed smokers (identified during follow-ups and IM conversation) will receive the "Advise" and "Refer" intervention.

NRT-S
One-week free NRT (gum or patch) will be provided with dosage based on time to first cigarette smoking after waking up in the morning and previous NRT use (standard practice and according to specific NRT product instructions). Subjects who have first cigarette <30 minutes in the morning or have previously used NRT, will receive 4mg nicotine gum or 21mg nicotine patch. Those who have first cigarette >30 minutes after waking up and have not previously used NRT will receive 2mg nicotine gum or 14mg nicotine patch. NRT use and potential side effects will be briefly explained orally based on standardized script according to the product instructions [12]. An NRT use card containing reminders of NRT use and potential side effects will be given

(Appendix 3).

IM Apps and Chatbot

Twelve-week personalized behavioural support will be delivered using interactive communication technologies using (1) regular tailored messages on abstinence, and (2) synchronousIM Apps conversation with trained SC advisors, and (3) a Chatbot. Their design will be guided by the Social Cognitive Theory (SCT) and Transtheoratical Model (TTM), which have been used in previous text-messaging-based SC trials [19, 20]. SCT posits that personal factors (cognitive, affective and biological events) and environmental factors affect behavioural changes. TTM postulates that smokers undergo 5 stages of change (pre-contemplation, contemplation, preparation, action, and maintenance or relapse) to achieve abstinence. Details of the intervention are as below:

(1) Regular tailored messages based on the subjects' surnames, socio-demographic characteristics (gender, occupation), smoking habit at baseline (nicotine dependence level, readiness to quit) and updated smoking status (smoking, quitting or relapsed) obtained during IM Apps conversation will be delivered through IM Apps (SCT). For smokers who set a quit date at baseline or during subsequent conversation through IM Apps, reminder messages will be sent before the quit date, followed by motivational messages that encourage abstinence to prevent relapse (TTM).

(2) IM Apps conversation with trained SC advisors is an extension of our baseline face-to-face AWARD intervention. Trained SC advisors will provide synchronous, personalized, interactive psychosocial support through IM conversation to help smokers to walk through the quitting process. Importantly, IM Apps allow advisors to provide timely responses to smokers' messages. These include providing support to avoid or handle high risk situations of smoking (e.g. cigarette invitation from friends, stressful events, boredom); and to break the habitual smoking by timely event-oriented messages (e.g. first cigarette in morning, smoking after meals, smoking during breaks at work). Advisors will also periodically proactively send IM messages in addition to regular messages described in (1) to initiate the conversation (e.g. asking recent progress of SC) and deliver evidence-based advice guided by the SCT and TTM. Advisors will actively refer smokers, if they have expressed the need, to SC services providers. A standard operation algorithm for SC advisor used in our pilot feasibility trial (ClinicalTrial.gov ID: NCT03182790) will be modified for use.

(3) A Chatbot will be built using open source NLP and machine intelligence platforms by the principal investigator (with knowledge and skills to be acquired through training and attachment to Harvard and MIT) and co-investigators (co-I's; Prof Kwok and Dr Kwok, computer engineers with rich experiences in developing interactive Chatbot for various purposes). In order to train the Chatbot to recognise Cantonese language (such as "點樣可以戒煙 ah?", "D 煙好貴"), a sequence-to-sequence neural network learning model will be developed. Our previous study (ClinicalTrials.gov ID: NCT03182790) on using WhatsApp to provide behavioural support for SC has recorded the conversation between over 200 smokers and advisors for 3 months. The unstructured text data will be used to train the proposed Chatbot (Appendix 4 shows examples of the text messages). Based on our previous extensive experience on SC counselling and the above-mentioned unstructured data, we will draft responses for each identified question (see Appendix 5 for examples), which will be further refined according to comments from experienced counsellors in TWGHs Integrated Smoking Cessation Center (ISCC, the largest SC service in HK, co-I Ms Chan). The completed responses algorithm will be reviewed and revised by smokers attending ISCC, SC counsellors and nurses. The prototype will then be pilot-tested in 5 smokers recruited from SH and will be revised according to their comments. The final version will be incorporated with Application Programming Interface (API) integration into user-friendly apps (iOS/Android/web app) with a backend server support and continuous data collection for potential bigdata analysis.

<u>Control groups</u>

The Control group will receive the same AWARD intervention as Intervention group at baseline but without NRT-S. At follow-up, they will receive regular SMS messages with content on general health and reminding the importance of participating in the follow-up surveys and biochemical validation for quitting. Our previous trial found regular SMS message on general health did not affect quitting [2].

(3) Study Design

Randomization and allocation concealment

Block randomization with blocks of 4/8/12 in random order will be used to individually allocate subjects into intervention or control groups with equal size. To conceal SC advisors from the random allocation sequence, a co-I (DYTC, a statistician) will prepare 664 identical, A5-size, sequentially numbered, opaque, sealed, envelopes (SNOSE). Each envelop will contain a card indicating the subject's allocation. Once a smoker has signed the consent form, an SC advisor will open one SNOSE according to the serial number to determine the group allocation. To avoid intervention contamination, each intervention will be delivered to one smoker at a time. Upon the completion of the first intervention, there will be a 5-minute interval to let the recruited smoker leave, before the next recruitment of a new smoker at the same SH. This approach will minimise the chance of the recruiting smokers who have a connection with the previous subjects.

(4) Data processing and analysis

Outcomes

The primary outcome is CO-validated (<4 ppm) smoking abstinence at 6-month and 12-month follow-up, which are the gold standard to determine abstinence in many SC trials [21, 22]. Secondary outcomes include self-reported 7-day point prevalence and continuous (24-week) abstinences, quit intention and attempts, smoking reduction (self-reported reduction in number of cigarettes per day by at least 50% of the baseline amount [23], calculated with inclusion and exclusion of quitters), nicotine addiction level (Heaviness Smoking Index), NRT and SC service use at 6- and 12-month. Change in quality-adjusted life year (QALY) will be estimated using the validated Chinese five-level EuroQol five-dimensional questionnaire (EQ-5D-5L) measured at baseline and 12-month to determine the incremental cost-effectiveness ratios of the intervention when compared to control (please see statistical analysis) [24]. Outcome assessors conducting the follow-up surveys will be blinded to the group allocation.

Measurements

Data (see table) will be collected at 3-, 6- and 12-month after recruitment using telephone surveys, each with an incentive of HK$50 cash coupon (total $150). Face-to-face exhaled CO validation tests using Smokerlyzer will be conducted at 6- and 12-month. HK$300 will be provided to subjects who completed each CO validation to compensate for travel expenses and time (total $600). Our experiences show that such incentives can substantially increase the response rate. Tablet PCs installed with Computer Assisted Patient Interview (CAPI) system will be used to collect data. The CAPI has built-in functions against errors and can easily generate datasets for progress monitoring and analysis, thus reducing the costs of data entry and cleaning. An electronic questionnaire using brief (<2 minutes) and validated questions (which have been used in many of our previous trials and by others) will be designed to measure the outcomes, and to maintain a higher retention rate and applicability in real-world practice. The questionnaire content is summarized as followed:

	Baseline	3M	6M	12M
Socio-demographic characteristics& smoking[1]	✓			
Smoking, quitting and reduction behaviors[2]	✓	✓	✓	✓
Self-efficacy, cessation service, NRT use[3]	✓	✓	✓	✓
CO validation of quit			✓	✓
Quality of life (EQ-5D-5L), medical service use	✓			✓

[1] Age, education level, year of smoking.[2] Number of cigarette consumed daily and time to first cigarette upon waking up in the morning (to calculate Heaviness Smoking Index), quit intention (yes/no), number of previous quit attempts. Past 7-day and continuous (24-week) smoking abstinences. Smoking reduction intention and attempts.[3] Recent use (past 24-week) of cessation medication, counselling, hotline, clinics, other SC programs.

Statistical and qualitative analysis

Intention-to-treat (ITT: most conservative approach in RCT) analysis will be used.

The **primary analysis** includes:

(1) Main effect: Intervention vs. Control on biochemically validated abstinence at 6-month and 12-month

The **secondary analyses** include:

(2) Main effect adjusting for baseline difference

(3) All secondary outcomes (see above) at 6- and 12-month

(4) Mediation analysis of 3-month factors (psychosocial or resource effects) on biochemically validated abstinence at 6-month (and 3- and 6-month factors on 12-month outcomes)
(5) Subgroup analyses based on intention to quit at baseline, SC service /NRT use at 3-month
(6) Cost-effectiveness analysis over the 12-month trial period and long-term cost-effectiveness analysis over the lifetime horizon (please see below)
(7) Qualitative study among quitters and non-quitters to understand the effects of the intervention
(8) Text-mining analysis of semi-structured and unstructured data from Chatbot and IM Apps

Sensitivity analyses (e.g. complete case or per-protocol) will be conducted, depending on the actual pattern of missing data, with different methods of statistical imputation (e.g. multiple/simple imputations/Last Observation Carried Forward) to assess the robustness of the findings. For the outcomes from multiple time points (3-, 6- and 12-month), linear mixed models, which allow for multiple observations between subjects and within subjects, will be used. Main effect and interaction effect will be included. Structural equation modelling will be used to assess direct and indirect effect of potential mediators (including self-efficacy on quitting, perceived support and SC services use) on outcomes. The content of Chatbot and IM Apps conversations will be recorded and analysed using skills acquired from the training (with assistance from co-Is) such as sentiment analysis, term frequency and topic modelling, which are common methods used in Bigdata analysis for text data.

Cost-effectiveness analysis of the intervention using standard methods by will be conducted by PI with assist from a co-I (Dr CK Wong, a health economist) [25]. For short-term cost-effectiveness, the empirical RCT data will be used to evaluate the effect in 12-month period. An ingredient approach will be used to estimate the cost of the intervention program including intervention materials (e.g. leaflet), administration fee, and time for SC advisors to deliver the intervention, whereas the healthcare resource use with respect to general and specialist outpatient visits, length of hospital stay, emergency visits will be measured for each subject in the Intervention and Control groups. The health effectiveness outcomes will include the number of quitters at 12-month and QALY gained. EQ-5D-5L utility scores at baseline and follow-up assessments will be used to construct QALY using area under the receiver operating characteristic curve approach. For long-term cost-effectiveness simulation to model the lifetime effect, a Markov model will be developed using the 12-month quit rate and relapse rate estimate taken from the RCT, and annual transition probabilities of smoking-related morbidities (e.g. COPD, lung cancer, stroke) taken from the literature. A perspective of healthcare provider will be taken and 3% discount rate will be applied. Treatment costs for smoking-related morbidities will be extracted from the literature. Setup and ongoing costs of the intervention and healthcare costs in both intervention and control groups will be derived from the RCT. Incremental cost-effectiveness ratios in the form of incremental cost per incremental life-year gained or QALY gained from intervention will be calculated over the lifetime of the simulated cohort. Deterministic and probabilistic sensitivity analyses will be conducted to test the robustness of the model.

A qualitative approach will be adopted for understanding experience of subjects in the intervention group by a Co-I (Dr JJ Lee, a qualitative researcher). The subjects will be recruited from subgroups based on smoking status at 12-month (quit, not quit) and Chatbot/IM Apps conversation involvement (>10 or <10 times). Semi-structured, individual interview will be conducted on at least 20 (N=5 for each subgroup) subjects with study endpoint determined by data saturation. All interviews will be audio-recorded and transcribed verbatim. Transcripts will be analyzed using thematic analyses in NVivo 11. Codes, categories and themes generated will be compared with the established taxonomy for evaluating intervention quality related to behavioural change technique for SC.

Training of SC advisors & intervention fidelity

10-15 undergraduates from health care disciplines will be trained as SC advisors in 1-day workshop. Which includes knowledge and skills related to SC, and specific skills on IM Apps use and responses by experienced IM researchers. We have extensive experience (more than 300 trained) on short and intensive SC training in many of our previous SC projects. Those who pass the written test will be qualified as an SC advisor. All the procedures will be clearly instructed using standard operation protocols. 10% of the recruitment sessions in the early phase of the trial will be randomly selected for checking for intervention fidelity using standardized forms. Experienced SC counselor will standby as a back up to handle any questions and problems by

telephone or IM Apps during recruitment. All IM Apps conversation will be recorded and 5% will be randomly checked (with no prior notification) for quality control.

[4000 words]

E. Key References

1. Census and Statistics Department. Thematic Household Survey Report No 59. Hong Kong: Hong Kong Special Administrative Region Government; 2016. Available from: http://www.digital21.gov.hk/eng/statistics/download/householdreport2016_59.pdf
2. Chan SS, Wong DC, Cheung YT, Leung DY, Lau L, Lai V, Lam TH. A block randomized controlled trial of a brief smoking cessation counselling and advice through short message service on participants who joined the Quit to Win Contest in Hong Kong. Health Educ Res. 2015;30(4):609-21.
3. Wang MP, Li WCH, Jiang N, Chu LY, Kwong A, Lai V, Lam TH. E-cigarette awareness, perceptions and use in community recruited smokers in Hong Kong. PLoS One. 2016;10(10):e0141683.
4. Chan SS, Cheung YT, Wan Z, Wang MP, Lam TH. Proactive and Brief Smoking Cessation Intervention for Smokers at Outdoor Smoking "Hotspots" in Hong Kong. J Cancer Educ. 2016. DOI: 10.1007/s13187-016-1085-3 [Epub ahead of print]
5. Wang MP, Li WCH, Cheung DY, Wu Y, Lam OB, Kwong AC, Lai VW, Chan SS, Lam TH. Brief advices on smoking reduction vs. abrupt quit for smoking cessation in Chinese smokers: a cluster randomized controlled trial. Nicotine Tob Res. 2017;20(1):67-72.
6. Cheung YT, Wang MP, Li HC, Kwong A, Lai V, Chan SS, Lam TH. Effectiveness of a small cash incentive on abstinence and use of cessation aids for adult smokers: A randomized controlled trial. Addict Behav. 2017;66:17-25.
7. Chan SSC, Cheung YTD, Wong YMB, Kwong A, Lai V, Lam TH. A Brief Smoking Cessation Advice by Youth Counselors for the Smokers in the Hong Kong Quit to Win Contest 2010: a Cluster Randomized Controlled Trial. Prev Sci. 2017. DOI: 10.1007/s11121-017-0823-z. [Epub ahead of print]
8. Cheung YTD, Lam TH, Li WHC, Wang MP, Chan SSC. Feasibility, Efficacy and Cost Analysis of Promoting Smoking Cessation at Outdoor Smoking "hotspots": a Pre-post Study. Nicotine Tob Res. 2017. DOI: 10.1093/ntr/ntx147. [Epub ahead of print]
9. Wang MP, Suen YN, Li HC, Lam C, O., Wu SY, Kwong AC, Lai VW, Chan SS, Lam TH. Intervention with brief cessation advice plus active referral for proactively recruited community smokers: a pragmatic cluster randomized clinical trial. JAMA Intern Med. 2017;177(12):1790-1797.
10. Lam TH, Abdullah AS, Chan SS, Hedley AJ. Adherence to nicotine replacement therapy versus quitting smoking among Chinese smokers: a preliminary investigation. Psychopharmacology. 2005;177:400-8.
11. Cummings KM, Fix B, Celestino P, Carlin-Menter S, O'Connor R, Hyland A. Reach, efficacy, and cost-effectiveness of free nicotine medication giveaway programs. J Public Health Manag Pract. 2006;12(1):37-43.
12. Miller N, Frieden TR, Liu SY, Matte TD, Mostashari F, Deitcher DR, Cummings KM, Chang C, Bauer U, Bassett MT. Effectiveness of a large-scale distribution programme of free nicotine patches: a prospective evaluation. Lancet. 2005;365(9474):1849-54.
13. Abdullah AS, Hedley AJ, Chan SS, Lam TH. A randomized controlled trial of two different lengths of nicotine replacement therapy for smoking cessation. Biomed Res Int. 2013;2013:961751.
14. Whittaker R, McRobbie H, Bullen C, Rodgers A, Gu Y. Mobile phone-based interventions for smoking cessation. Cochrane Database Syst Rev. 2016;4:CD006611.
15. Scott-Sheldon LA, Lantini R, Jennings EG, Thind H, Rosen RK, Salmoirago-Blotcher E, Bock BC. Text messaging-based Interventions for smoking cessation: a systematic review and meta-analysis. JMIR Mhealth Uhealth. 2016;4(2):e49.
16. Hoermann S, McCabe KL, Milne DN, Calvo RA. Application of synchronous text-based dialogue systems in mental health Interventions: systematic review. J Med Internet Res. 2017;19(8):e267.

17. Hamine S, Gerth-Guyette E, Faulx D, Green BB, Ginsburg AS. Impact of mHealth chronic disease management on treatment adherence and patient outcomes: a systematic review. J Med Internet Res. 2015;17(2):e52.
18. Fitzpatrick KK, Darcy A, Vierhile M. Delivering cognitive behavior therapy to young adults with symptoms of depression and anxiety using a fully automated conversational agent (Woebot): a randomized controlled trial. JMIR Ment Health. 2017;4(2):e19.
19. Abroms LC, Boal AL, Simmens SJ, Mendel JA, Windsor RA. A randomized trial of Text2Quit: a text messaging program for smoking cessation. Am J Prev Med. 2014;47(3):242-50.
20. Naughton F, Cooper S, Foster K, Emery J, Leonardi-Bee J, Sutton S, Jones M, Ussher M, Whitemore R, Leighton M, Montgomery A, Parrott S, Coleman T. Large multi-centre pilot randomized controlled trial testing a low-cost, tailored, self-help smoking cessation text message intervention for pregnant smokers (MiQuit). Addiction. 2017;112(7):1238-49.
21. Stead LF, Perera R, Bullen C, Mant D, Hartmann-Boyce J, Cahill K, Lancaster T. Nicotine replacement therapy for smoking cessation. Cochrane Database Syst Rev. 2012; CD000146.
22. Stead LF, Hartmann-boyce J, Perera R, Lancaster T. Telephone counselling for smoking cessation. Cochrane Database Syst Rev. 2013;CD002850.
23. Lam TH, Cheung YT, Leung DY, Abdullah AS, Chan SS. Effectiveness of smoking reduction intervention for hardcore smokers. Tob Induc Dis. 2015;13(1):9.
24. Luo N, Liu G, Li M, Guan H, Jin X, Rand-Hendriksen K. Estimating an EQ-5D-5L value set for China. Value Health. 2017;20(4):662-9.
25. Cromwell J, Bartosch WJ, Fiore MC, Hasselblad V, Baker T. Cost-effectiveness of the clinical practice recommendations in the AHCPR guideline for smoking cessation. JAMA. 1997;278:1759-66.

Front:

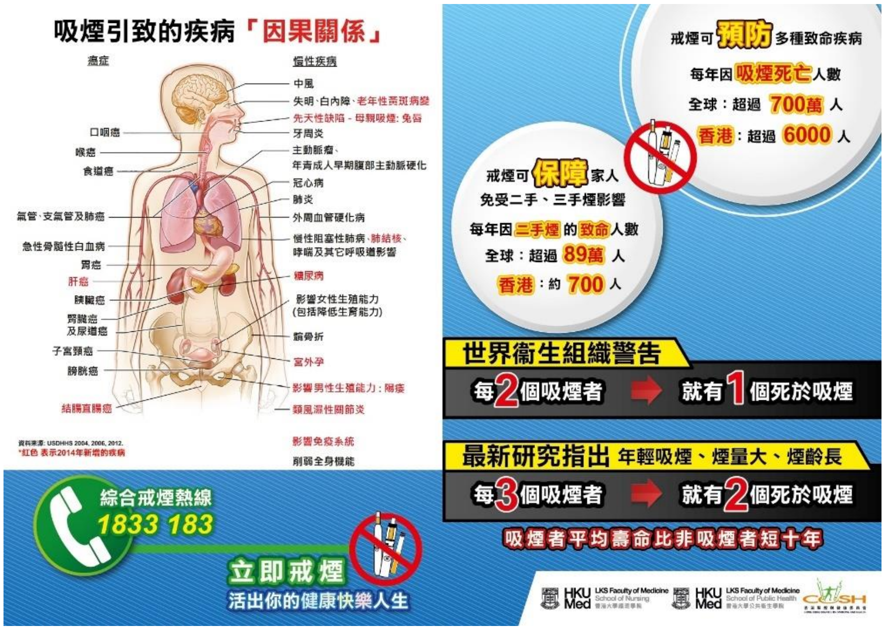

Back:

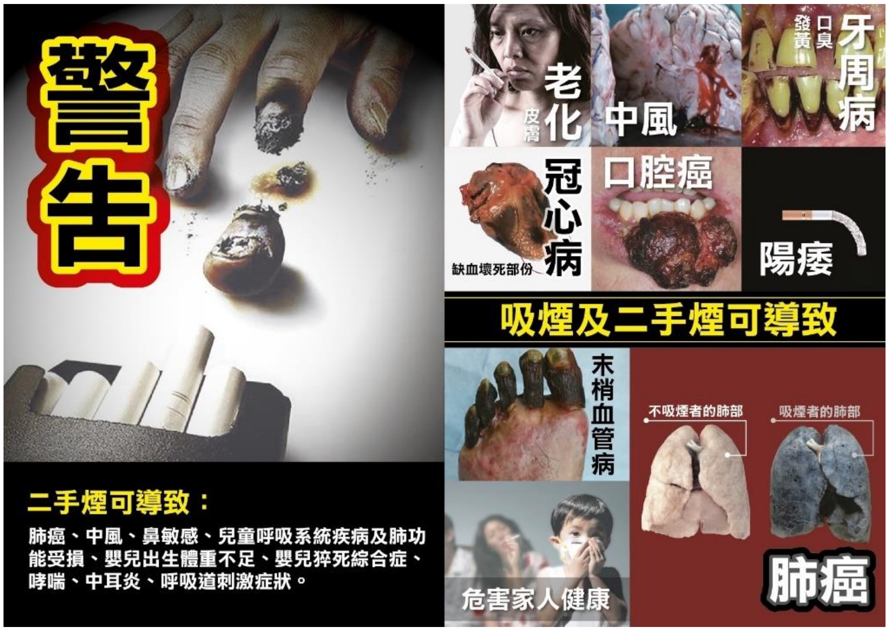

Figure 1. Health warning leaflet. Front contents included tobacco-related mortality and diseases caused by smoking. Back contents included health effects of cigarette smoking and secondhand smoking.

Front:

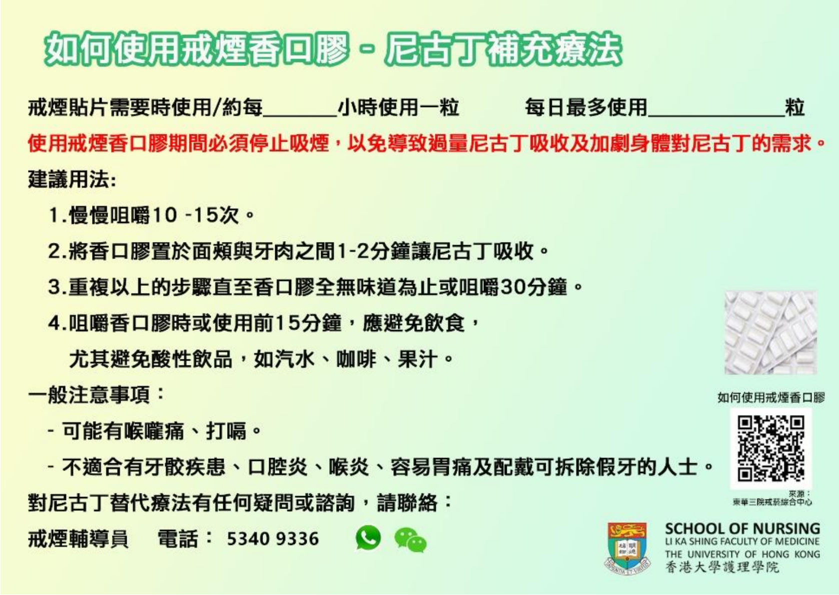

Back:

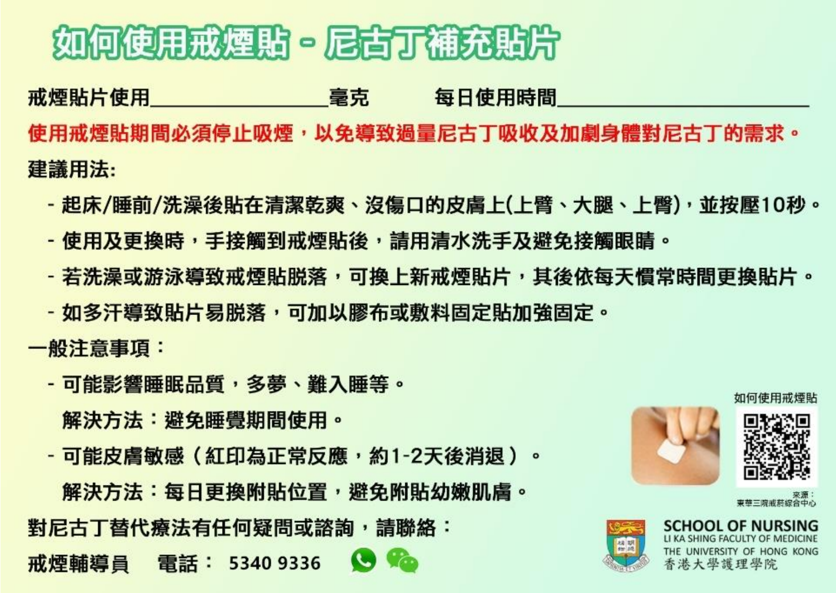

Figure 2. Instruction card of nicotine replacement therapy. Front contents included methods of use nicotine gum, such as chewing the gum slowly for about 10-15 times, parking the gum between your cheek and the ivory inlaid bed for about 1-2 minutes, repeating these steps for about 30 minutes until the taste becomes faded, etc. Back contents included methods of use nicotine patch, such as applying a new patch each day to non-hairy sites of your body including arms, back, and abdomen, may still use the patch when you swim or have showers, etc.

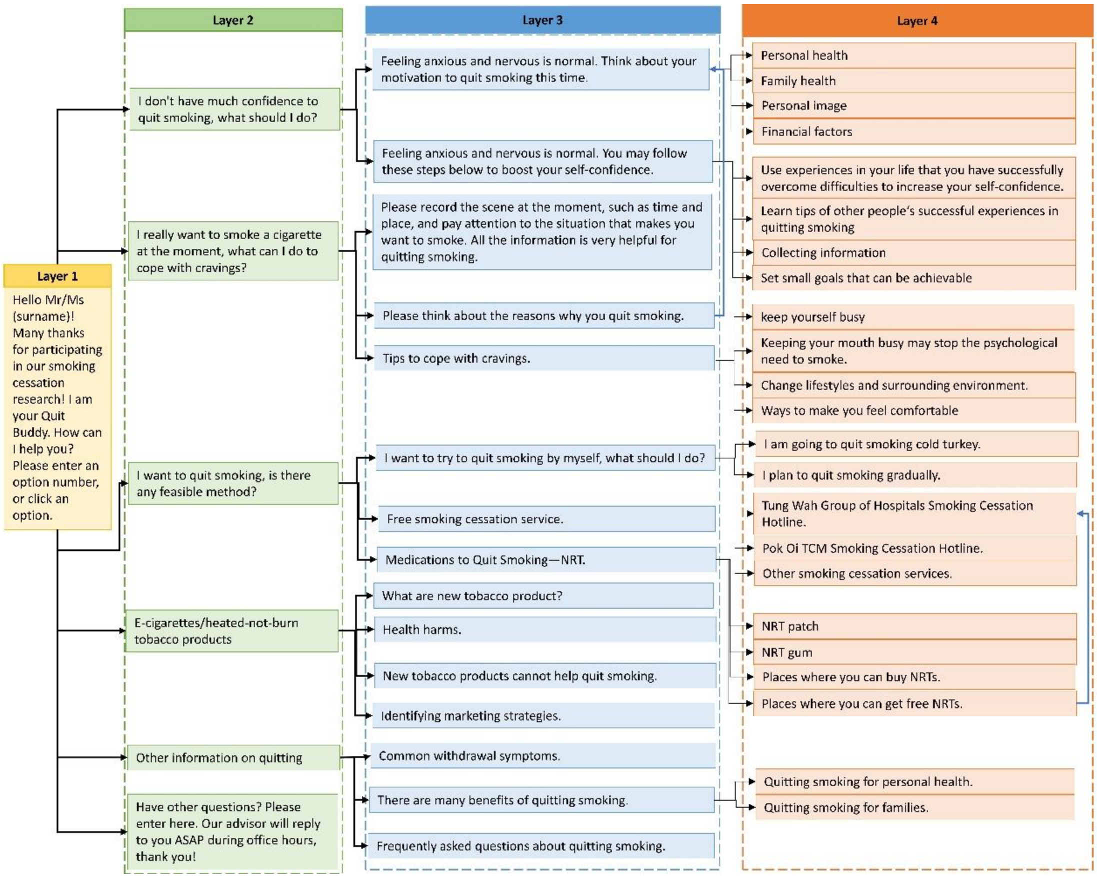

Figure 3. Layered structure of the chatbot. Note: The structure of the chatbot included 5 layers. Layer 5, which refers to detailed responses to specific sub-themes in layer 4, are not presented due to the length limit. Example response in layer 5 can be found in Table 1 below.

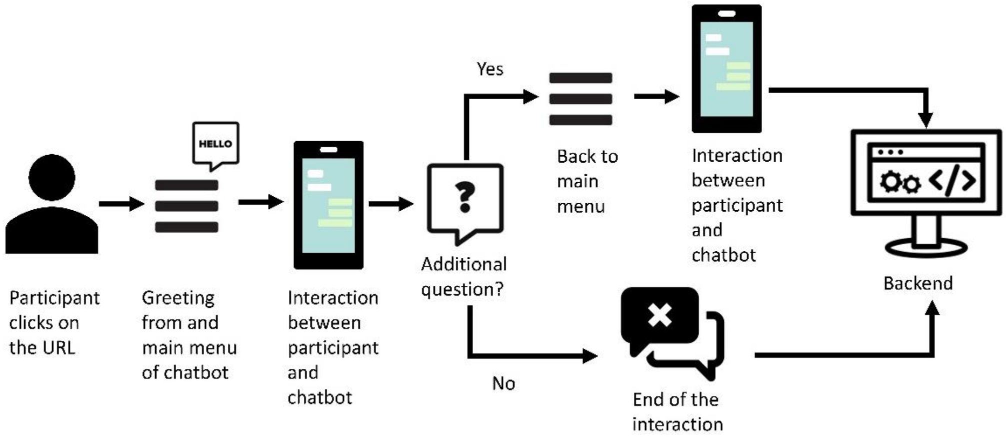

Figure 4. Schematic of how the systems of the chatbot interact

Table 1. Contents of the chatbot

Theme	Subtheme	Example response
**Quitting methods**	Local smoking cessation services	Tung Wah Group of Hospitals Integrated Center on Smoking Cessation can provide tailored treatment plan for you. You can apply their service through calling the quitline at 23328977.
	Medications	How to use nicotine patch? You can apply a new patch each day to arms, back, or abdomen.
	Quitting smoking on one's own	Try lengthening the interval between each smoking and reduce numbers of cigarettes smoked each day.
**Coping with cravings**	Identification of specific cues and contexts	Please tell me about your current location, such as near your home, company, etc. Or if someone close by is smoking right now.
	Reinforcement of quit motivations	Quitting smoking can protect the health of you and your family.
	Tips to reduce cravings	Try changing your morning routine (such as the order of breakfast and bathing).
**Improving self-efficacy**	Reinforcement of quit motivations	Quitting smoking can save money.
	Strategies to improve confidence	You may have tried successfully overcoming difficulties at work or persevering in learning a new language. You can apply these successful experiences to smoking cessation actions!
**New tobacco products**	Introduction to the products	E-cigarettes are new tobacco products in which aerosol is delivered by heating a liquid that usually contains nicotine.
	Health effects	The harm of e-cigarettes should not be underestimated. E-cigarettes can produce harmful substances and carcinogens after heating and vaporizing chemical substances at high temperatures.
	Marketing strategies	Be careful! The packaging of most e-cigarettes may mislead consumers with words such as "not addictive", "certified", and "environmental protection."
**Other frequently asked questions**	-	Withdrawal symptoms such as stress and irritation are common. Have a chat with your friends is a good way to change your current mental state.
**Input textbox**	-	Please enter your question. Our smoking cessation advisors will reply to you as soon as possible during office hours. Thank you!

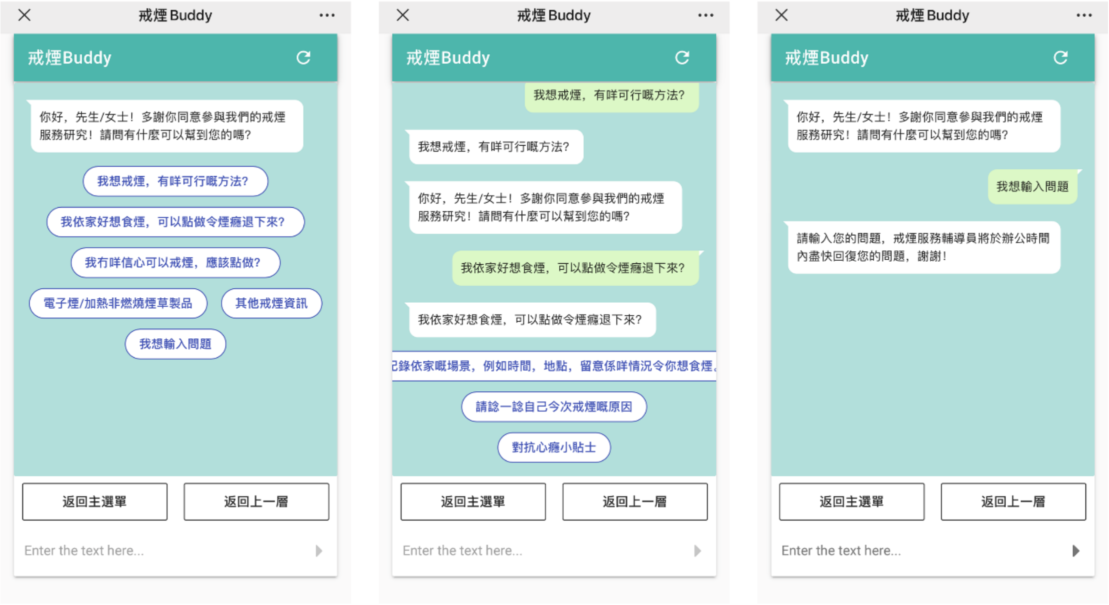

A B C

Figure 5. Screenshots of the chatbot "Quit Buddy". Interface of the main menu (A) allows participants to click into themes on quitting methods, coping with cravings, improving self-efficacy, new tobacco products, other frequently asked questions, and an input textbox. Interface of the example theme "coping with cravings" (B) allows participants to interact with subthemes on identification of specific cues and contexts, reinforcement of quit motivations, and tips to reduce cravings. Interface of the input textbox (C) allows participants to make enquires to live smoking cessation advisors.

Table 2. Sensitivity analyses of smoking cessation outcomes based on complete case and multiple imputations (N=664)

	Complete case		Multiple imputation	
	OR (95% CI)	P	OR (95% CI)	P
**Primary outcomes**				
Validated abstinence				
6 months	1.34 (0.58, 3.13)	0.49	1.22 (0.53, 2.82)	0.64
12 months	1.22 (0.60, 2.47)	0.59	1.14 (0.56, 2.33)	0.72
**Secondary outcomes**				
Self-reported 7-day point-prevalent abstinence				
6 months	1.19 (0.69, 2.05)	0.53	1.09 (0.65, 1.84)	0.74
12 months	1.07 (0.64, 1.80)	0.79	1.14 (0.68, 1.92)	0.61
Self-reported 24-week continuous abstinence				
6 months	0.91 (0.49, 1.72)	0.78	0.91 (0.48, 1.72)	0.78
12 months	0.88 (0.49, 1.59)	0.67	0.88 (0.49, 1.59)	0.67
Smoking reduction by at least 50% of baseline[a]				
6 months	1.17 (0.76, 1.82)	0.48	1.12 (0.73, 1.71)	0.61
12 months	1.33 (0.89, 1.99)	0.16	1.34 (0.90, 2.00)	0.15
Quit attempt				
6 months (cumulative)	1.51 (1.05, 2.17)	0.026	1.37 (0.98, 1.91)	0.068
12 months (cumulative)	1.38 (0.96, 1.99)	0.08	1.24 (0.88, 1.77)	0.22
Use of smoking cessation service				
6 months (cumulative)	1.64 (0.92, 2.95)	0.10	1.66 (0.94, 2.94)	0.08
12 months (cumulative)	1.34 (0.81, 2.19)	0.25	1.39 (0.86, 2.25)	0.18

OR: odds ratio; CI: confidence interval.

[a] Quitting not included as reduction.

Table 3. Side effects of NRT-S use by symptom in the intervention group

	Total (n=85)	Gum (n=21)	14 mg patch (n=26)	21 mg patch (n=38)
No side effect	57 (67.1)	10 (47.6)	20 (76.9)	27 (71.1)
Skin problems	7 (8.3)	0	1 (3.9)	6 (15.8)
Oral/throat problems	2 (2.4)	1 (4.8)	1 (3.9)	0
Headache/dizziness	10 (11.8)	3 (14.3)	3 (11.5)	4 (10.5)
Stomach problems (e.g. indigestion, hiccups)	4 (4.7)	4 (19.1)	0	0
Insomnia problems	2 (2.4)	0	1 (3.9)	1 (2.6)
Change in taste	3 (3.5)	3 (14.3)	0	0
Breathing problems	0	0	0	0
Heart problems (e.g. palpitations, chest pain)	0	0	0	0

© 2023 Guo N. et al.
